# On the computational assessment of white matter hyperintensity progression: difficulties in method selection and bias field correction performance on images with significant white matter pathology

**DOI:** 10.1007/s00234-016-1648-3

**Published:** 2016-01-30

**Authors:** Maria del C. Valdés Hernández, Victor González-Castro, Dina T. Ghandour, Xin Wang, Fergus Doubal, Susana Muñoz Maniega, Paul A. Armitage, Joanna M. Wardlaw

**Affiliations:** Department of Neuroimaging Sciences, Centre for Clinical Brian Sciences, Chancellor’s Building, 49 Little France Crescent, Edinburgh, EH16 4SB UK; College of Medicine and Veterinary Medicine, University of Edinburgh, Edinburgh, UK; Department of Cardiovascular Sciences, University of Sheffield, Sheffield, UK

**Keywords:** MRI, Cerebrovascular disorders, Leukoencephalopathies, White matter hyperintensities, Neuroimaging

## Abstract

**Introduction:**

Subtle inhomogeneities in the scanner’s magnetic fields (B_0_ and B_1_) alter the intensity levels of the structural magnetic resonance imaging (MRI) affecting the volumetric assessment of WMH changes. Here, we investigate the influence that (1) correcting the images for the B_1_ inhomogeneities (i.e. bias field correction (BFC)) and (2) selection of the WMH change assessment method can have on longitudinal analyses of WMH progression and discuss possible solutions.

**Methods:**

We used brain structural MRI from 46 mild stroke patients scanned at stroke onset and 3 years later. We tested three BFC approaches: FSL-FAST, N4 and exponentially entropy-driven homomorphic unsharp masking (E^2^D-HUM) and analysed their effect on the measured WMH change. Separately, we tested two methods to assess WMH changes: measuring WMH volumes independently at both time points semi-automatically (MCMxxxVI) and subtracting intensity-normalised FLAIR images at both time points following image gamma correction. We then combined the BFC with the computational method that performed best across the whole sample to assess WMH changes.

**Results:**

Analysis of the difference in the variance-to-mean intensity ratio in normal tissue between BFC and uncorrected images and visual inspection showed that all BFC methods altered the WMH appearance and distribution, but FSL-FAST in general performed more consistently across the sample and MRI modalities. The WMH volume change over 3 years obtained with MCMxxxVI with vs. without FSL-FAST BFC did not significantly differ (medians(IQR)(with BFC) = 3.2(6.3) vs. 2.9(7.4)ml (without BFC), *p* = 0.5), but both differed significantly from the WMH volume change obtained from subtracting post-processed FLAIR images (without BFC)(7.6(8.2)ml, *p* < 0.001). This latter method considerably inflated the WMH volume change as subtle WMH at baseline that became more intense at follow-up were counted as increase in the volumetric change.

**Conclusions:**

Measurement of WMH volume change remains challenging. Although the overall volumetric change was not significantly affected by the application of BFC, these methods distorted the image intensity distribution affecting subtle WMH. Subtracting the FLAIR images at both time points following gamma correction seems a promising technique but is adversely affected by subtle WMH. It is important to take into account not only the changes in volume but also in the signal intensity.

**Electronic supplementary material:**

The online version of this article (doi:10.1007/s00234-016-1648-3) contains supplementary material, which is available to authorized users.

## Introduction

The presence of hyperintensities on T2-weighted and fluid attenuated inversion recovery (FLAIR) brain magnetic resonance imaging (MRI) in white and deep grey matter regions is a common neuroradiological finding in non-diseased older people [1] and people with neurological disease [2]. They are commonly known as white matter hyperintensities (WMH) or white matter lesions [2] and appear on computed tomography as areas of decreased density and on MRI T1-weighted images as regions of decreased signal intensity when compared to the normal-appearing white matter tissue.

These hyperintensities also represent a significant proportion of the burden of pathology in the brains of patients with neurological diseases, [1, 2] and their progression has been associated with atherosclerosis, [3] high blood pressure, [4] stroke [5, 6] and dementia [5, 7]. Therefore, their assessment criteria and methods to study their progression have attracted significant attention, [8] and it is of increasing clinical interest to have reliable and practical techniques for quantification of WMH in routine neuroradiological practice. So far, visual rating scales and semi-automatic thresholding of FLAIR images have been the main methods used to assess WMH changes [8] (see Supplementary Table S1 for a review). Visual scales are practical and quick but considered to be prone to observer variation. Semi-automated or automated computational methods are seen to be more sensitive and reproducible, although the manual correction that is currently required by most computational methods is time consuming, introduces subjectivity, and a preferred method has not yet emerged. Nonetheless, numerous companies, including MR scanner manufacturers, are developing automated WMH quantification methods. It is therefore incumbent on the neuroradiologist to understand the basis, scope and limitations of these techniques.

The use of different thresholding criteria when assessing WMH has led to inconsistencies in study results [9] and motivated the development of other WMH change quantification approaches based on intensity differences and/or morphological tissue transformations [10, 11]. These methods either use a subtraction pipeline to detect WMH changes [10] or consider the mass effect of the WMH in the surrounding tissue to determine structural changes in the vicinities of the WMH detected at baseline [11]. Simultaneous analysis of images obtained at different time points could potentially reduce the errors produced by independent assessments. But whether or not and, if so, how the presence of ill-defined subtle T2-weighted/FLAIR hyperintensities alters the performance of this type of analysis has not yet been reported. These diffuse and non-continuous white matter hyperintensities, with varying erratic intensity patterns emerging from the lateral ventricle walls [12–15], have been considered an indicator of pre-lesional changes [13] and have received attention as it appears they indicate subtle tissue damage due to an inflammatory process or neurodegeneration [12]. These subtle WMH occur in addition to regular “high intensity” WMH and are typically excluded by previous methods that measure WMH volume as a single entity [16]. Our findings suggested that a closer agreement with the visual ratings performed by trained neuroradiologists could be obtained by improving computational detection of subtle WML [16].

However, irrespective of the method used, the assessment of WMH is affected by non-uniform transmit/receive B_1_ fields, generated by the RF coils during the MR scanning process, that results in a low frequency corruption of signal intensity values across the image [10, 17, 18]. Therefore, algorithms that attempt to correct for the effects of B_1_ inhomogeneities are routinely included as part of some computational image analysis approaches (See Supplementary Table S1). Progression of white matter disease is commonly reported by the assessment of WMH independently at each time point using the same method (i.e. assessment criteria) (Supplementary Table S1) and using detailed protocols aimed at reducing false hyperintensities or artefacts that can confound accurate identification [19, 20]. Thus, it is imperative to test first the individual effect of the bias field correction (BFC) algorithms to, then, explore how it translates to the end result (i.e. longitudinal assessment of WMH change) in a computational pipeline.

**Fig. 1:**
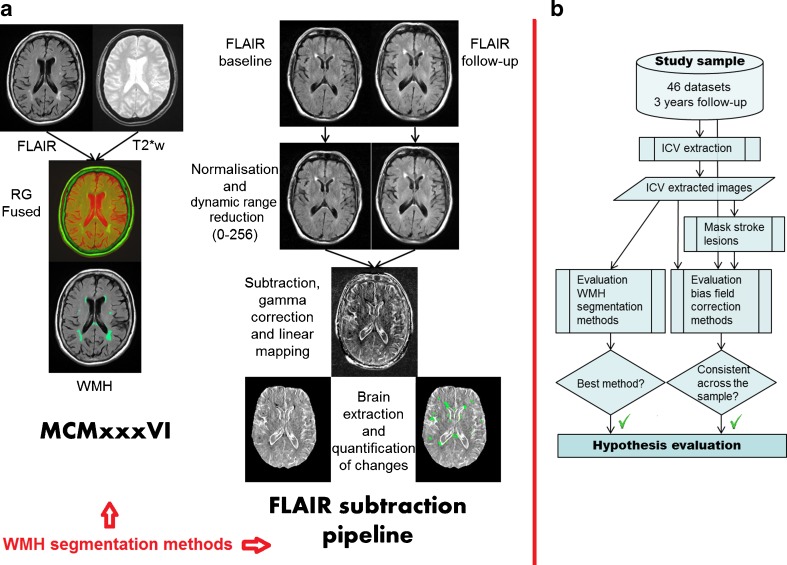
Workflow of the WMH segmentation methods (**a**) and pipeline to evaluate the hypothesis that correcting for B_1_ inhomogeneities can alter the assessment of WMH progression (**b**)

The main aim of this paper is to raise awareness on the implications that applying a BFC method have for patient monitoring not just clinical research and evaluate the performance of the computational methods that are part of a pipeline to assess WMH change. For this, we, first, evaluate the effect that three state-of-art BFC methods, commonly used as part of these pipelines, have on WMH change and give recommendations on how to proceed when their use is required. Second, we evaluate two intensity-based approaches that measure WMH change: one representative of the group of techniques that quantify WMH volume separately at each time point and another representative of the methods that use subtraction pipelines. Finally, we compare the measurements obtained from applying the winning method of assessing WMH progression with and without the winning BFC method to illustrate how much results can differ (Fig. 1).

From the different techniques that measure WMH change, we avoided approaches that use deformation fields to quantify structural changes, since these do not consider the mass effect of the WMH, and hence do not detect static lesions [11] (i.e. lesions that remain unchanged). Both selected methods are semi-automatic, use thresholding and are followed by manual removal of false positives and stroke lesions after careful and individual inspection. To facilitate the generalisability of our findings, none of the two techniques applied require a training set to derive the parameters used to perform their task, which could bias the accuracy of the results towards datasets similar to those used in our study.

## Materials and methods

### Subjects and brain MRI acquisition

We used brain MRI datasets from 46 patients (11 women) with lacunar (*n* = 22) or mild cortical (*n* = 24) stroke who were recruited prospectively in a study of stroke mechanisms [21]. Patients were scanned on two occasions: median 12 days (IQR 4–27 days) after presenting to hospital with acute stroke symptoms and after a median of 39 months later (IQR 30–45 months). The mean age at baseline was 66 years (SD ±10). The median baseline National Institute of Health Stroke Scale score of all patients that provided imaging data for the present analyses was 2 (IQR 1–3). Formal written consent from all subjects and ethical approval were acquired.

MRI was conducted in the Brain Research Imaging Centre, University of Edinburgh (http://www.bric.ed.ac.uk). A GE Signa Horizon HDx 1.5 T clinical scanner (General Electric, Milwaukee, WI), equipped with a self-shielding gradient set and manufacturer-supplied eight-channel phased-array head coil, was used to acquire structural brain FLAIR (TR/TE/TI 9002/147/2200 ms, 240 × 240 mm FOV, 256 × 256 acquisition matrix), T2-weighted (TR/TE 5000/140 ms, 240 × 180mm FOV, 256 × 256 acquisition matrix) and T2*-weighted (TR/TE 620/15 ms, 240 × 180 mm FOV, 256 × 192 acquisition matrix) all with 5-mm slice thickness, 1.5-mm inter-slice gap and voxel size of 0.94 × 0.94 × 6.5 mm [3]. Calibration sequences, magnet shimming and visual quality assurance were performed during each scanning session, and sequence parameters were the same at baseline and follow-up.

### Image analysis

We generated binary masks of the intracranial volume (ICV) from the T2*-weighted images [22] and new and old stroke lesions from the FLAIR and T2-weighted images following standard protocols [23] (see Online Methods). We assessed WMH volume changes using two methods (Fig. 1a): (1) quantifying WMH volume independently at baseline and follow-up using MCMxxxVI [24, 25] (www.sourceforge.net/projects/bric1936), a multispectral thresholding-based technique, and (2) subtracting post-processed FLAIR baseline from follow-up images [10] (see Online Methods for details) and assessed their performance by visual inspection of the WMH change masks. We annotated the number of false positives and partial and total false negatives per region produced by each WMH change quantification method. Then, we selected the method that performed best to evaluate the effect that correcting the MRI images for B_1_ magnetic field inhomogeneities had on WMH change.

### Correction of MRI images for B_1_ magnetic field inhomogeneities

We tested the step of compensating for slow-varying image intensity gradients through an adaptive low-pass filtering technique, as it is often used in image processing pipelines. For this, we selected two publically available and widely used methods: N4 (http://www.slicer.org/slicerWiki/index.php/Documentation/4.3/Modules/N4ITKBiasFieldCorrection) [26] and FSL-FAST (http://fsl.fmrib.ox.ac.uk/fsl/fslwiki/FAST)[27]. We also tested a third method: the 3D exponentially entropy-driven homomorphic unsharp masking (E^2^D-HUM), [17] that reported having similar performance but without requiring tuning of its parameters nor any a-priori assumptions about the tissues. E^2^D-HUM has been implemented in a grid infrastructure [18]. We evaluated the performance of these three BFC methods using (a) images without any pre-processing, (b) images after ICV extraction and (c) ICV extracted images after removing the stroke lesions.

All BFC image processing methods, whilst correcting for spatial low-frequency variations, skew the intensity distributions of each tissue type, facilitating the differentiation between tissue classes. To evaluate how much the measurements of WMH volume change could be affected by each of the BFC methods tested and derive guidelines to minimise this effect, we conducted the experiments summarised in Table 1. We applied the BFC method that performed more consistently across imaging modalities and across tests to assess whether correcting the images for inhomogeneities has any effect in the measurement of WMH changes (Fig. 1b, Table 2).

**Table 1:** Tests to evaluate the BFC methods’ performance on the sample. Description, rationale and expected outcome

Test no.	Test description	Rationale and expected outcome
1	(a) Segment (i.e. extract) the ICV on FLAIR images. (b) Apply the 3 BFC methods to the “ICV extracted” images. (c) Apply minimum variance quantisation to original and BFC FLAIR “ICV extracted” images using 5 quantisation levels. (d) Compute the spatial differences in levels’ boundaries at baseline and follow-up on regions that changed and on those that remained unchanged.	This quantisation method optimises the clusterisation of the image intensity levels. The quantised levels correspond to: Level 1: cerebrospinal fluid and background, Level 2: partial volume effect between cerebrospinal fluid and brain tissue, Level 3: normal-appearing brain parenchyma, Level 4: subtle WMH, Level 5: intense WMH. As each quantised level gathers voxels within minimum intensity differences, the BFC method that introduces less distortion will be the one that causes the spatial distribution of the voxels from levels 3 and 4 to be more similar to the one obtained without applying any BFC method.
2	(a) Segment (i.e. extract) the ICV on T2W and T2*W images. (b) Apply the 3 BFC methods to the “ICV extracted” images. (c) Determine the difference between the variance-to-mean ratio (ΔVMR) on the regions occupied by normal tissues in the “ICV extracted” original T2W, T2*W and FLAIR and their respective BFC images. (d) Analyse ΔVMR between baseline and follow-up and between corrected and uncorrected images.	Differences in the coefficient of variation, similar metric to the VMR, have been previously used to evaluate the performance of BFC methods [17, 29] If the VMR, as normalised measure of dispersion of the intensities in the normal tissues, is similar before and after BFC (i.e. ΔVMR_baseline_ → 0 and ΔVMR_follow-up_ → 0), then the BFC method most likely preserves better the original image intensity distribution. This will also be the case if the ΔVMR_original_ ≈ ΔVMR_corrected_. If, on the contrary, ΔVMR_baseline_ → max and ΔVMR_follow-up_ = → max, the BFC method reduced the intra-class variance with respect to its mean, facilitating the tissue segmentation most probably at expenses of distorting the subtle intensity differences within the tissue class. Hyperintensities were excluded so as to increase the sensitivity of the test for subtle intensity changes.
3	Visually inspect the results: bias field patterns and T2W, T2*W and FLAIR BFC images with respect to the original (i.e. non-BFC) images.	The bias field pattern recognised by a good BFC method will not depend on whether the skull and the stroke lesions are previously removed from the image or not.

**Table 2:** Tests to evaluate the WMH change assessment methods’ performance on the sample and the effect of BFC on the winner method. Description, rationale and expected outcome

Purpose	Tests’ description	Rationale and expected outcome
Evaluate the output and performance of the computational methods for calculating WMH volume change.	(1) Annotate the performance of each method (without BFC) on each dataset on the brain regions specified by the Prins scale [29], brainstem and cerebellum and summarise the results of the visual inspection.	The best method should be robust against artefacts and accurately highlight zones of increase/decrease in WMH.
	(2) Calculate the correlation between the volume of WMH change by each method (without BFC) and the Prins visual rating scale. Cross-sectional results from MCMxxxVI are also evaluated against Fazekas scores as per [18].	The output from the best method should correlate highly and significantly with the output from the visual rating.
Evaluate the influence that the BFC has on the output of the winning computational method	(1) Calculate the correlation between the volume of WMH change obtained by the winning method with and without BFC (the latter done also with the winning method) and the Prins visual rating scale. If the winning method is MCMxxxVI, cross-sectional results are also evaluated against Fazekas scores as per [18].	If the application of BFC is beneficial, the correlation between the output of the WMH volume change measurements when this is applied and the visual ratings should be higher and stronger than when the BFC is not applied.
	(2) Calculate the correlation between the volume of WMH change obtained by the winning method with and without BFC (the latter done also with the winning method) and age.	If the application of BFC is beneficial, the correlation between the output of the WMH volume change measurements when this is applied and age should be higher and stronger than when the BFC is not applied.
	(3) Visually inspect the performance and results of the winning computational method when BFC images are used vs. those obtained without the previous application of this step (i.e. BFC).	If the application of BFC is beneficial, the results should not differ significantly from those obtained when the original images are used, and the manual correction to the automatically obtained results should be minimal.

### Other statistical analyses

We explored the volumetric agreement between BFC methods’ results using Bland-Altman analysis [28] and plotted the Jaccard similarity index against the mean values of the volumetric measurements [25]. IBM SPSS Statistics v21 was used to calculate the descriptive statistics of the WMH change with each method. Significant differences between the results obtained from each procedure described above were determined by the related-samples Wilcoxon signed rank test. In absence of a “ground truth” or “gold standard reference” with which to compare the results from each method, a detailed visual assessment of the performance of each BFC and WMH change assessment method on regions of interest was also performed, as explained above, to help decide which method performed best.

The correlation between computational methods’ results and those obtained from the visual rating scales [18, 29] (see Online methods) and between the computational output from the preferred method—before and after BFC—and age, were calculated using the Robust Correlation MATLAB Toolbox [30]. Normality was evaluated using the Henze-Zirkler Multivariate Normality Test [31]. As WMH change computational measurements were not normally distributed (*p* value associated to the Henze-Zirkler statistic < 0.02 in all cases), and heteroscedastic, they were rescaled and log-transformed for computing their correlation with age and visual ratings.

## Results

The highest sensitivity and best performance in our sample, determined visually after repeatedly applying the three methods to T2W, T2*W and FLAIR baseline images with different parameters, was obtained with five and six classes for FSL-FAST, the default parameters for N4 and the cut-off frequency of the low-pass Butterworth filter equal to 0.001 for E^2^D-HUM. The selection of 5–6 tissue classes when applying FSL-FAST was determined where the resultant “segmented” image showed distinct “real” tissue/abnormalities subdivisions as closely as possible; notwithstanding, tissue segmentation on our sample was not accurate by this method despite trying several combinations of input parameters. All BFC methods were run on an Intel ® Xeon® E5-2665 processor at 2.40 GHz with 20 MB cache size. The time for processing a single image was 1.5 min for FSL-FAST, between 20 and 60 s for N4 and approximately 2 s for E^2^D-HUM.

### Effect of the correction for spatial intensity variations (BFC)

#### Test 1—BFC methods: analysis of the 5-level grey-scale quantised images

1. From the three BFC methods evaluated, E^2^D-HUM preserved best the spatial distribution of the subtle and more intense regions (which corresponded to intensity levels 4 and 5 respectively). N4 preserved less the original intensity distributions as indicated in Fig. 2.

**Fig. 2:**
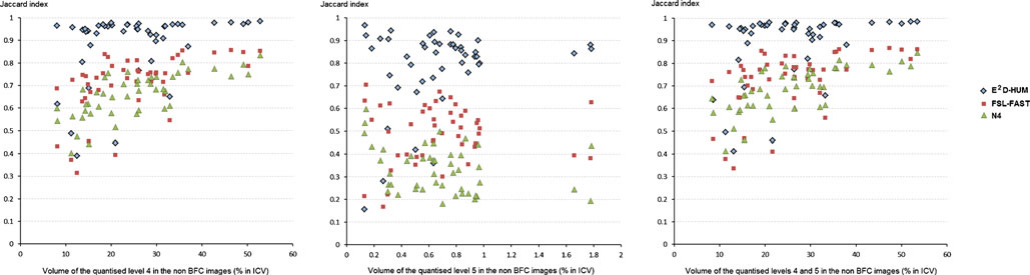
Modified Bland-Altman plots of the spatial agreement between the levels 4 and 5 (i.e. subtle and more intense regions respectively) of the 5-level grey scale quantised baseline FLAIR images before and after BFC by each method. The *horizontal axes* represent the number of voxels of the quantised levels on the images without BFC. The *vertical axes* represent the Jaccard index
2. None of the BFC methods reduced the “top/bottom hat” intensity effect from the images: the subtle intensities on the upper and bottom slices appeared always overestimated (Supplementary Fig. S1) at the centre compared with those at the borders and in other slices, with concentric and gradual attenuation towards the borders.
3. All BFC methods alter the spatial intensity distribution, but in most slices/datasets these variations are small and are not visually noticeable (Supplementary Fig. S1).
4. From the quantised images, the total volume change of the subtle and more intense regions (quantised levels 4 and 5) between the two time points obtained with BFC from FSL-FAST had better agreement with that obtained without BFC (mean difference 0.009 % of ICV, 95 % CI [−5.16, 5.14]), than any other method: for N4 it was 1.40 % of ICV, 95 % CI [−6.72, 3.92] and for E^2^D-HUM it was 0.65 % of ICV, 95 % CI [−3.78, 5.09] (Supplementary Fig. S2).
5. The total volume of the subtle and more intense regions (quantised levels 4 and 5) that remained unchanged after 3 years obtained with N4 had better agreement with that obtained without BFC (mean difference −1.54 % of ICV, 95 % CI [−7.02, 3.93]) than any other method: for FSL-FAST it was −3.72 % of ICV, 95 % CI [−12.96, 5.53] and for E^2^D-HUM it was −2.11 % of ICV, 95 % CI [−11.14; 6.91] (Supplementary Fig. S3). However, whilst FSL-FAST and E^2^D-HUM performed quite consistently for most cases, the agreement obtained between the quantised images before and after BFC using N4 was biassed: mean differences were high when the unchanged volume of the quantised hyperintensities was small and very low (negative values) when it was extensive.

#### Test 2—BFC methods: differences between the VMR on normal tissues

The VMR differences (ΔVMR) between time points and BFC vs. uncorrected FLAIR, T2*W and T2W are provided in a supplementary table (Table S2). The smallest difference in the VMR of intensities, measured on normal tissues, between BFC and uncorrected images was obtained with E^2^D-HUM applied to FLAIR after extracting the ICV: 0.08 (IQR 0.10) for baseline and 0.16 (IQR 0.07) for follow-up images. The biggest difference was obtained when the three methods were applied to the original follow-up T2-weighted images (i.e. without previous extraction of the ICV or stroke lesion): 36.28 (IQR 15.79) with FSL-FAST, 16.35 (IQR 7.45) with N4 and −3.42 (IQR 2.67) with E^2^D-HUM (Table S2). However, the results from the analysis of ΔVMR were generally good and consistent with all the three methods, these being significant across all tests for FSL-FAST.

#### Test 3—BFC methods: visual inspection of the BFC images

**Fig. 3:**
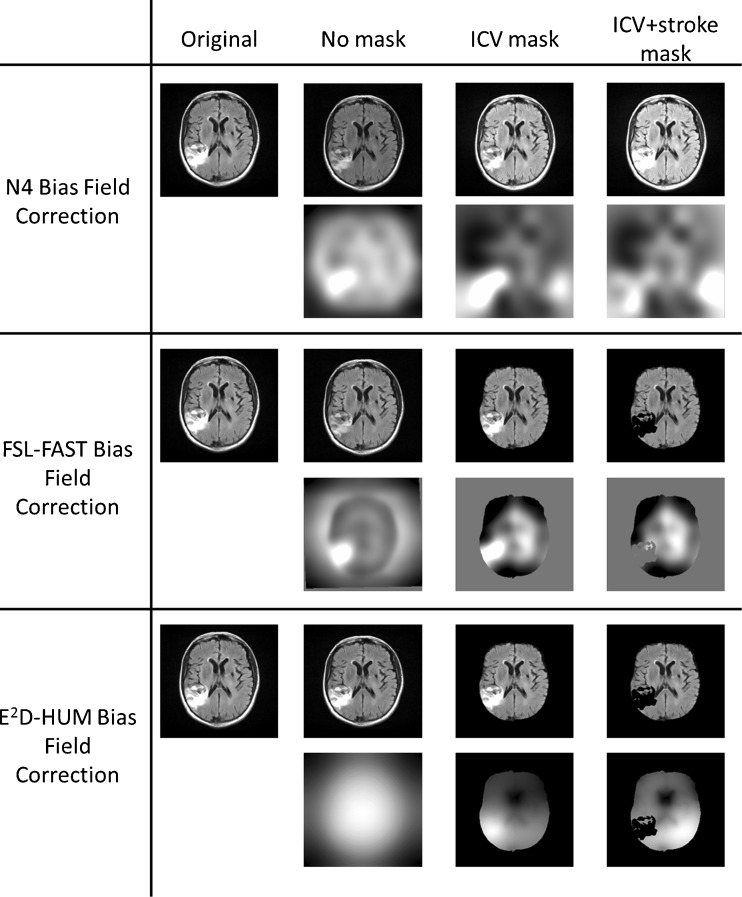
Example of the performance of the BCF methods on the FLAIR images. All images have the same levels of brightness and luminance. On the *top row* are the original vs. corrected images (i.e. after applying a BFC technique) the *bottom row* shows the correspondent bias field maps estimated from each case

We performed visual evaluation of maps of the bias of the magnetic field obtained with each method, the original images and the BFC images (Fig. 3 main text and Figs. S4 and S5 in the Supplements). We found that in datasets with confluent WMH and/or medium-sized to large cortical lesions:

1. In FLAIR images, the hyperintensities were attenuated in the regions where they were prominent. The pattern of WMH distribution was slightly distorted as all BFC methods were sensitive to these medium-sized to large lesions (Fig. 3),
2. In FLAIR images, FSL-FAST and E^2^D-HUM preserved more consistently the original intensity levels of the normal-appearing tissues, as opposed to N4 (see top row of each method on Fig. 3),
3. FLAIR was the modality in which the estimated bias field was more influenced by large or confluent lesions. This was not the case for T2- or T2*-weighted (Supplementary Figs. S4 and S5 vs. Fig. 3),
4. The bias field estimated from FSL-FAST did not change depending on whether or not the ICV and the stroke lesion were extracted (see Fig. 3, bottom row for each method). This visual analysis agrees with the numerical analyses of the VMR differences shown in Table S2. For E^2^D-HUM, when the ICV and the stroke lesion are not extracted, the BFC algorithm estimates a perfect “disc” (see bottom row of Fig. 3), and when the stroke lesion is extracted, it estimates an increase in the bias field on the contralateral hemisphere (this is for FLAIR and T2-weighted).

### Evaluation of the WMH change assessment methods in absence of BFC

The WMH median volume change over 3 years obtained with MCMxxxVI was 2.9 ml (IQR = 7.4). These measurements differed significantly (*p* < 0.001) from those obtained from the subtraction pipeline (median = 7.6 ml, IQR = 8.2).

#### Test 1—WMH change assessment methods: visual inspection of the output

The subtraction of the post-processed FLAIR images was more robust than MCMxxxVI avoiding artefact effects in regions where they are common: bilateral Sylvian fissures and insular cortex, vicinities of the fornix, third and fourth ventricle, aqueduct and cistern ventral to mesencephalon, amygdaloid nucleus, anterior temporal poles and pathways of the corticospinal tracts. However, the presence of subtle WMH considerably influenced the outcome from this method inflating the result: regions of subtle WMH at baseline that, after 3 years, became strongly hyperintense on the FLAIR scans, were also counted as part of the volumetric change (i.e. increase) (Fig. S6). In addition, the FLAIR subtraction pipeline quantified together the tissue loss due to atrophy (i.e. that was not a WMH at baseline) and the WMH that disappeared and corresponded to tissue loss at follow-up (i.e. enlarged ventricles). Overall, a quantitative volumetric evaluation was not possible: the regions identified as “increase” in WMH volume by the subtraction method were not spatially coincident with those identified by the multispectral method (Fig. S6) and visually there was an increase in the signal intensity on all those regions anyway, but of a different degree.

#### Test 2—WMH change assessment methods: correlation between the output of the WMH change assessment methods and visual ratings

The volumetric results from the FLAIR subtraction method significantly correlated (*p* = 0.002) with Prins visual ratings (Spearman *ρ* = 0.435, CI = [0.180 0.646]). The correlation slightly strengthened when outliers were removed (Spearman *ρ* = 0.463, CI = [0.210 0.675]). The volumetric results obtained from MCMxxxVI correlated weakly with the Prins visual ratings: Spearman *ρ* = 0.126, CI = [−0.151 0.407]. However, the correlation between the WMH volumes obtained at each time point with Fazekas scores was strong and significant (*p* < 0.0001) before and after removing outliers (Spearman *ρ* = 0.549, CI = [0.244 0.789] (before) and Spearman *ρ* = 0.740, CI = [0.518 0.882] (after outliers’ removal)).

### Effect of BFC on WMH volume change

Given the results from the previous subsections, we selected the results from FSL-FAST to evaluate the effect of BFC on WMH volume measurement using MCMxxxVI.

**Fig. 4:**
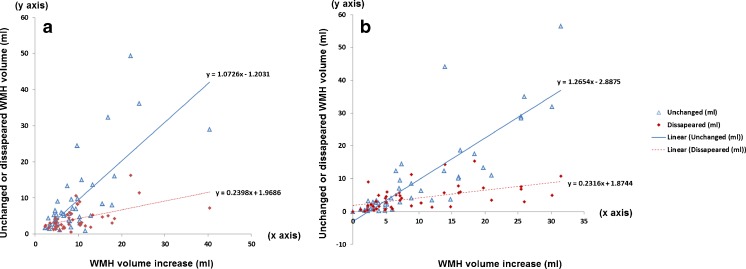
Relationship between total WMH volume increase after 3 years and volumes of WMH that remained unchanged or disappeared assessed using MCMxxxVI. **a** Using images after correcting for magnetic field inhomogeneities using FSL-FAST and **b** using images without this post-processing step

1. Total WMH gross volume change (WMH volume at follow-up—WMH volume at baseline)
  The WMH median volume change was 2.9 ml (IQR = 7.4) when the images were used without BFC and 3.2 ml (IQR = 6.3) when a preliminary BFC step was introduced. These measurements did not differ significantly (*p* = 0.544).
2. WMH volume that increased, decreased and remained unchanged at follow-up (spatial differences in volume change)
  The general pattern of WMH change obtained using MCMxxxVI differed across the sample when the images were BFC (Fig. 4a) compared with when the original images were used (Fig. 4b). However, the proportion of WMH that increased, disappeared or were unchanged after 3 years was almost the same regardless of the introduction of this step (observe the equations of the trend-line for each case in Fig. 4).

#### Test 1—effect of BFC on the performance of the computational method: correlation between the output of MCMxxxVI with and without BFC and visual ratings

The correlation between the WMH volume change and Prins visual ratings strengthened when BFC images were used: Spearman *ρ* = 0.126, CI = [−0.151 0.407] (without BFC) and Spearman *ρ* = 0.280, CI = [−0.047 0.543] (with BFC). However, the correlation between the cross-sectional WMH volume measurements and Fazekas scores, although remaining significant (P < 0.0001), weakened when BFC images were used: Spearman *ρ* = 0.549, CI = [0.244 0.789] (without BFC) and Spearman *ρ* = 0.478, CI = [0.158 0.738] (with BFC).

#### Test 2—effect of BFC on the performance of the computational method: correlation between the output of MCMxxxVI with and without BFC and age

The correlation between the WMH volume change and baseline patient’s age was significant (*p* = 0.014) when measurements were done using the original images (i.e. without BFC): Pearson’s *r* = 0.222, CI = [0.005 0.433], but became weaker and not significant (*p* > 0.05) when BFC images were used: Pearson’s *r* = 0.140, CI = [−0.086 0.348].

#### Test 3—effect of BFC on the performance of the computational method: visual inspection

WMH in the inferior brain stem and cerebellum were mostly under-detected with MCMxxxVI when BFC was not applied and most accurately detected otherwise. In the same way, artefactual hyperintensities in the vicinities of the Sylvian and midsagittal frontal fissures (in the axial plane) were incorrectly included within the initial WMH mask and had to be removed manually afterwards in the absence of BFC. They were, however, correctly undetected when the BFC was applied prior to the WMH segmentation. In general, BFC considerably reduced to minimal the amount of manual editing after the automatic WMH segmentation. However, when the resultant WMH binary mask was superimposed into the original images, some small punctate WMH in the deep white matter were erroneously undetected in an irregular and indistinct manner. In addition, the boundaries of the correctly identified WMH did not always correspond to the “real” WMH boundaries.

## Discussion

Computational methods that process neuroradiological images produce data that is used for individual patient monitoring and as key evidence in clinical research. Our results indicate that introducing a step of correcting the FLAIR images for apparent inhomogeneities in the B_1_ magnetic field influences the quantitative assessment of WMH on each individual dataset and therefore the assessment of its change over time. The correlation of the neuroradiological visual assessments with the computational measurements of WMH volume and WMH progression is also affected by the application of BFC methods. However, the overall proportion of WMH volume that increases, decreases and disappears at follow-up with respect to baseline may not be affected by the application of a BFC technique if it proves to be consistent across the sample regardless or not of the presence of a hyper/hypo-intense mass (e.g. a stroke lesion or a tissue loss due to an old stroke). Of note, the delineation of the stroke lesion should always be done in the original images (i.e. without applying any BFC method) because all BFC methods tested reduce its size as they over-attenuate its intensity. Previous studies of longitudinal WMH change that have corrected MR images for inhomogeneities in the magnetic field have applied these methods to healthy ageing individuals or patients with diseases known to exhibit patterns of diffuse distribution of WMH (Supplementary Table S1), contrary to the datasets evaluated in this study which have a prominent mass of hyperintense tissue and regions of various extents with ill-defined less-intense WMH. Quantifying the WMH volume at both time points using a thresholding-based technique might be a good approach for cross-sectional analyses, but for longitudinal evaluations, a detailed quantitative and qualitative analysis of the signal strength on regions in which the WMH observed at baseline newly appear or disappear is recommended.

As the quality of the BFC that FSL-FAST performs is heavily dependent on the quality of the segmentation, prior to evaluating this method, we optimised the number of tissue classes checking that the segmentations were reasonable. However, this was not achieved for brains with high and low load of WMH, and neither for brains affected by large cortical strokes in which T2W/FLAIR hyperintensities have appreciable mass effect. In such conditions, FSL-FAST did not separate well the tissues and the BFC rather seemed to try equalising out their intensities. However, from the three BFC methods applied, FSL-FAST gave more consistent results as it distorted less the intensity levels and estimated similar bias field on images with and without masking ICV and/or the stroke lesion (i.e. better results from tests 2 and 3, see Tables 1 and 2). Other studies for which FSL-FAST had not performed well, [17] concluded that when the effect of the inhomogeneities is low, like it is in our sample, FSL-FAST had had the best performance.

Rather than evaluating the BFC methods per se, we evaluated their effect on the spatial intensity distribution of our datasets to investigate their possible effect on the quantification of WMH change. In our view, our merit lays on providing: (1) a methodology to evaluate the performance of BFC methods on image intensities and, in turn, on WMH quantification and (2) evidence of the possible effect of BFC methods on the quantitative assessment of WMH change. Each BFC method estimated the “bias field” differently. As the “ground truth” of the bias field is unknown, they are generally evaluated on synthetic images. In practice, quality control MRI scanning protocols and improvements on the MR scanner and coil manufacture contribute to reduce bias field inhomogeneities. Clinical studies are more likely to have images similar to the ones used for this study rather than to the synthetic images used to validate the BFC methods. Therefore, our results are more likely to represent the “real world” situation. Despite these techniques performing differently in the presence and absence of T2W/FLAIR hyperintensities’ mass effect (e.g. multiple sclerosis patients vs. patients with microvascular disease), the methodology proposed here is generalizable as we carefully selected a sample with a wide range of variation in the load, pattern and distribution of WMH and, in general, of T2W/FLAIR hyperintensities.

The use of a multispectral approach on the subtraction pipeline, suggested and tested previously [12], has been reported to reduce false detected regions while increasing the sensitivity for detecting WMH change. It would be interesting to reproduce these tests incorporating also T1-weighted images to explore whether the BFC methods affect the outcome of this approach and if so, to what degree. Nevertheless, as for the detection of WMH, FLAIR is a must-use sequence and it is considerably affected by the BFC image processing methods, we would recommend not applying any BFC technique to this image modality. For the rest imaging modalities, the performance of various BFC methods across the sample should be evaluated before any is applied, to guarantee consistency in the results.

## Conclusions

This paper gives an insight and raises awareness on an issue to improve upon the way current analysis of WMH progression is being conducted. Quantification of WMH changes is important for assessing the progression/regression of various CNS disorders. Quantification may be used for individual monitoring thus affecting clinical decisions per patient as well as for studying disease and drug mechanisms of action on various patient populations. Still reliable quantification of WMH and their evolution may be hampered by false hyperintensities or artefacts induced by magnetic field inhomogeneities which may vary between acquisition systems and individual patients. An attempt, however, to correct for these undesirable effects, may be accompanied by the distortion of the real hyperintensities if careful evaluation and analysis of the image processing BFC method in the specific imaging datasets to be studied is not done beforehand. For this, the tests shown in Tables 1 and 2 of this paper are suggested, and in the presence of significant white matter pathology, it is recommended not to apply any image processing BFC procedure to the FLAIR MRI modality. The use of image subtraction pipelines for quantifying WMH change seems promising, but more research is needed to improve their sensitivity to subtle intensity changes. WMH quantification techniques should take into account not only the changes in volume but also in the signal intensity.

## Electronic supplementary material

Below is the link to the electronic supplementary material.

ESM 1 (DOCX 31 kb)

Table S1 (DOCX 25 kb)

Table S2 (DOCX 16 kb)

Fig. S1 (JPG 644 kb)

High Resolution Image (TIF 1648 kb)

Fig. S2 (JPG 93 kb)

High Resolution Image (TIF 122 kb)

Fig. S3 (JPG 99 kb)

High Resolution Image (TIF 122 kb)

Fig. S4 (JPG 223 kb)

High Resolution Image (TIF 727 kb)

Fig. S5 (JPG 244 kb)

High Resolution Image (TIF 780 kb)

Fig. S6 (JPG 179 kb)

High Resolution Image (TIF 700 kb)

## Abbreviations

WMH: White matter hyperintensities
FLAIR: Fluid attenuated inversion recovery
MRI: Magnetic resonance imaging
CI: Confidence interval
IQR: Interquartile range
BFC: Bias field correction
E^2^D-HUM: Exponentially entropy-driven homomorphic unsharp masking
FSL-FAST: FMRIB software library-FMRIB’s automated segmentation tool
MCMxxxVI: Multispectral colour mapping with variance identification
