# Supplementary material for: On the computational assessment of white matter hyperintensity progression: difficulties in method selection and bias field correction performance on images with significant white matter pathology
Source: Neuroradiology. 2016 Jan 30;58:475–85. doi: 10.1007/s00234-016-1648-3 (PMC4846712; doi:10.1007/s00234-016-1648-3)
Supplement: Supplementary file 1 — (DOCX 31 kb) [file 234_2016_1648_MOESM1_ESM.docx]

**On the computational assessment of white matter hyperintensity progression: difficulties in method selection and bias field correction performance on images with significant white matter pathology**

**Online Methods**

Image Segmentation of the Intracranial Contents

We segmented the intracranial volume (ICV) (i.e. contents within the inner skull table including brain tissue, cerebrospinal fluid, veins and dura) semi-automatically from the T2*-weighted images, considering an inferior limit on the axial slice just superior to the tip of the odontoid peg at the foramen magnum, We used the Object Extraction Tool in Analyze 11.0^TM^ (AnalyzeDirect Inc, Mayo Clinic) to automatically generate a binary mask of the ICV, and the Region-Of-Interest Tool in the same software to manually correct for any inaccuracies afterwards[1]. The brain extraction in the MRI images was performed by multiplying the original images by the ICV binary mask.

Masking of Stroke Lesions

The stroke lesions were extracted semi-automatically by thresholding of FLAIR images using a region-growing algorithm in Analyze 11.0 (module: “Region-Of-Interest”). New (i.e. index) stroke lesions were identified at baseline as the region on the FLAIR image corresponding to a hyperintense area on the averaged diffusion weighted image. Old cortical stroke lesions were identified as visible hyperintense regions on FLAIR and T2-weighted MRI extending to the cortex, generally bordering a distinguishable region of low density with negative mass effect. The binary masks of the stroke lesions obtained at baseline were also applied to the follow-up scans after co-registration using FSL-FLIRT (<http://fsl.fmrib.ox.ac.uk/fsl/fslwiki/FLIRT>).[2]

Computational Assessment of WMH Volume Changes

The two methods evaluated for assessing WMH volume changes used images rigidly and linearly co-aligned using FSL-FLIRT (<http://fsl.fmrib.ox.ac.uk/fsl/fslwiki/FLIRT>).[2]

1. Quantification of WMH volume independently at baseline and follow-up

We selected a multispectral thresholding-based method that has been validated against the conventional thresholding of FLAIR images[3] and other multispectral classifiers[4]: MCMxxxVI (Figure 1A in the main text). It has been applied to studies of aging,[5-7] stroke[8] and Alzheimer’s disease,[9] and is implemented on a tool freely available from ([www.sourceforge.net/projects/bric1936](http://www.sourceforge.net/projects/bric1936)). Briefly, WMH are automatically extracted on the quantised colour image obtained after co-registering FLAIR and T2*-weighted sequences, mapping them in green and red respectively, fusing them and applying minimum variance quantisation within the intracranial volume. Then, blinded to any clinical information, artefacts were removed manually as described in (<http://www.bric.ed.ac.uk/research/imageanalysis.html>). Index and old stroke lesions were manually excluded from the final WMH mask following the process described previously.

This process (i.e. WMH volume assessment) was done twice by the same observer on the subset of 46 datasets: firstly using the images after BFC and, then, using the images without this correction step. This method (i.e. to measure WMH volume independently at baseline and follow-up) permits the quantification of WMH changes in two ways: 1) as the gross volume difference between the WMH volumes measured at baseline and follow-up; and 2) as the spatial differences between the WMH binary masks obtained independently at baseline and follow-up (i.e. classifying the volume of WMH that disappeared becoming normal tissue or cerebrospinal fluid at follow-up, along with that which appeared and/or remained unchanged at follow-up).

b) WMH change determined from an image subtraction pipeline

We implemented a fairly standard image subtraction pipeline that consisted in subtracting previously co-registered normalised FLAIR images at both time points and enhancing the contrast of the difference image so as to facilitate the quantification of the white matter change (Figure 1A in the main text). It was accomplished using a tool named ‘longitudinal changes’ developed in-house. This tool first does normalisation and dynamic grayscale range reduction of each FLAIR volume (i.e. at baseline and follow-up) by dividing the intensity on each voxel by the maximum intensity value and rescaling it from 0 to 256 values. Then, it subtracts the resultant baseline FLAIR volume from the follow-up and does a linear intensity transformation on the resultant volume that involved 3 stages: normalisation, gamma correction and linear mapping [10]. This process can be represented by the equation:

where I_max_ and I_min_ are the maximum and minimum intensity levels of the input volume (i.e. resultant from the subtraction) and and are the final and initial intensity levels for a voxel identified by its coordinates (x,y,z). We used a gamma factor equal to 1 to enhance the contrast by saturating the 1% of the lowest and highest intensities at the minimum and maximum values respectively. Finally we applied the ICV mask and quantified, separately, the number of voxels that represent an “increase” in WMH volume (hyperintense), “decrease” in WMH and “tissue loss”. These last two are hypointensities that are distinguished from each other by a threshold level that needs to be manually entered at the discretion of the observer. The graphic interface unit of the software allows a post-processing step of manual editing to correct for errors that could occur due to artefacts.

Visual Assessment of WMH Changes

WMH changes were visually assessed using the Prins visual rating scale [11], which records the increase (1), no-change (0) and decrease (-1) of the WMH burden in frontal, lateral and occipital periventricular, and frontal, parietal, temporal and occipital deep regions. We summed the overall changes recorded by this scale on each region to generate a “WMH change score” that ranged from -7 to 7. All MRI datasets were assessed, independently, at baseline and follow-up using Fazekas visual ratings [7]. A total Fazekas score, ranging from 0 to 6, was obtained by summing the periventricular and deep white matter scores.

Reference List

1. Valdes Hernandez MC, Royle NA, Jackson MR, Munoz Maniega S, Penke L, Bastin ME, Deary IJ, Wardlaw JM (2012) Color fusion of magnetic resonance images improves intracranial volume measurement in studies of aging. Open Journal of Radiology 2: 1-9

2. Jenkinson M, Bannister P, Brady M, Smith S (2002) Improved optimization for the robust and accurate linear registration and motion correction of brain images. Neuroimage 17: 825-841

3. Hernandez MC, Ferguson KJ, Chappell FM, Wardlaw JM (2010) New multispectral MRI data fusion technique for white matter lesion segmentation: method and comparison with thresholding in FLAIR images. Eur Radiol 20: 1684-1691

4. Valdes Hernandez MC, Gallacher PJ, Bastin ME, Royle NA, Maniega SM, Deary IJ, Wardlaw JM (2012) Automatic segmentation of brain white matter and white matter lesions in normal aging: comparison of five multispectral techniques. Magn Reson Imaging 30: 222-229

5. Pohjasvaara T, Mantyla R, Salonen O, Aronen HJ, Ylikoski R, Hietanen M, Kaste M, Erkinjuntti T (2000) How complex interactions of ischemic brain infarcts, white matter lesions, and atrophy relate to poststroke dementia. Arch Neurol 57: 1295-1300

6. Hernandez MC, Piper RJ, Bastin ME, Royle NA, Maniega SM, Aribisala BS, Murray C, Deary IJ, Wardlaw JM (2014) Morphologic, distributional, volumetric, and intensity characterization of periventricular hyperintensities. AJNR Am J Neuroradiol 35: 55-62

7. Valdes Hernandez MC, Morris Z, Dickie DA, Royle NA, Munoz Maniega S, Aribisala BS, Bastin ME, Deary IJ, Wardlaw JM (2012) Close correlation between quantitative and qualitative assessments of white matter lesions. Neuroepidemiology 40: 13-22

8. Wang X, Valdes Hernandez MC, Doubal F, Chappell FM, Wardlaw JM (2012) How much do focal infarcts distort white matter lesions and global cerebral atrophy measures? Cerebrovasc Dis 34: 336-342

9. Mikula M, Proitsi P, Sattlecker M, O'Sullivan M, Simmons A, Mecocci P, Soininen H, Tsolaki M, Vellas B, Lovestone S, Hodges A (2013) Association of magnetic resonance imaging white matter lesions and cognitive decline in Alzheimer's disease. Alzheimers Dement 9: P701

10. Llado X, Ganiler O, Oliver A, Marti R, Freixenet J, Valls L, Vilanova JC, Ramio-Torrenta L, Rovira A (2012) Automated detection of multiple sclerosis lesions in serial brain MRI. Neuroradiology 54: 787-807

11. Prins ND, van Straaten ECW, van Dijk EJ, Simoni M, van Schijndel RA, Vrooman HA, Koudstaal PJ, Scheltens P, Breteler MMBBarkhof F (2004) Measuring progression of cerebral white matter lesions on MRI. Visual rating and volumetrics. Neurology 62:1533-1539
