# Supplementary material for: On the computational assessment of white matter hyperintensity progression: difficulties in method selection and bias field correction performance on images with significant white matter pathology
Source: Neuroradiology. 2016 Jan 30;58:475–85. doi: 10.1007/s00234-016-1648-3 (PMC4846712; doi:10.1007/s00234-016-1648-3)
Supplement: Supplementary file 2 — (DOCX 25 kb) [file 234_2016_1648_MOESM2_ESM.docx]

**On the computational assessment of white matter hyperintensity progression: difficulties in method selection and bias field correction performance on images with significant white matter pathology**

**Supplementary Table S1. Assessment of white matter hyperintensity (WMH) changes on freely accessible publications of longitudinal studies on WMH progression in the last 15 years (1999-2014)** (Results obtained from Medline database after removing duplicates and manuscripts from the same primary study if used the same method of assessing WMH changes. Search terms were: white matter hyperintensity + changes, longitudinal + white matter hyperintensities, white matter lesions + longitudinal, white matter lesion + changes)

| **Ref. no.** | **Study, sample size and type** | **Age at baseline (mean ± SD)** | **Follow-up period (mean ± SD)** | **Details of the assessment of WMH change** | | | **Bias-field inhomogeneities correction (Y/N)** | **MRI scan at baseline: magnet strength and sequences** | **Same scanning protocol at follow-up (Y/N)** |
| --- | --- | --- | --- | --- | --- | --- | --- | --- | --- |
|  |  |  |  | **Quantitative** | **Visual Rating** | **Separately at each time point (Y/N)** |  |  |  |
| 1 | 211 MS patients | 44±11 years | 1 year | WMH (MS lesions) semi-automatically delineated by thresholding using AMIRA 3.3.1 | N | Y | Not reported | 1.5T  Sequences: T1-weighted (MPRAGE) and proton density | Y |
| 2 | 225 individuals from the Tasmanian Study of Cognition and Gait (TASCOG) | 71.4 ± 6.8 years | 30.6 ± 4.9 months | Fully automated morphological watershed segmentation on FLAIR, T1- and T2-weighted followed by statistical classifiers to remove false positives. | N | Y | Y  Combination of filters from the Insight Toolkit framework | 1.5T  T1-, T2-weighted, FLAIR, gradient echo (GRE) | Y |
| 3 | 26 patients with cerebral Amyloid Angiopathy | 69.1 ± 6.5 years | Median 1.1 years | Signal intensity thresholding using MRIcro followed by manually editing | N | Y | N | 1.5T  Sequences: T1-, T2-weighted (some had FLAIR) | Y |
| 4 | 83 participants: 55 type 2 diabetes patients and 28 healthy controls | 65.9 ± 5.4 | 4.1 ± 0.4 years | WMH measured using k-nearest neighbour-based probabilistic segmentation | N | Y | Y  Based on histogram standardisation | 1.5T  Sequences: T1-, T2-weighted, IR, FLAIR | Not specified |
| 5 | 35 Alzheimer’s disease patients | 66.2 ± 8.6 years | 2.2 years | N | Rotterdam Scan Study visual rating scale on hard copy prints | Y | N | 1.0T  T1-weighted and proton density or FLAIR | Y |
| 6 | 50 Alzheimer’s disease patients and 45 elderly controls | 71.2 ± 8.1 years | 3 years | N | Visually rated on follow-up scans and reported changes with respect to baseline | N | N | CT scans | N (three different CT scanners were used) |
| 7 | 41 migraineurs | 40.5 ± 14.6 years | Mean 33.2 months | N | WMH counted visually: number present on both sequences: FLAIR and T2-weighted, and new WMH. | Y | N | 1.5T  Sequences: T2-weighted and FLAIR | Y |
| 8 | 845 participants of the Epidemiology of Vascular Ageing (EVA) Study | 59 to 71 years (65.01 ± 3.05 years) | 4 years | N | Scale from A to D: A no lesion; B mild; C moderate; D severe | Y | N | 1.0T  T1-, T2-weighted and proton density | Y |
| 9 | 133 hypertensive individuals from the Study on Cognition and Prognosis in the Elderly (SCOPE) | 70-89 years | 2 years | FLAIR images used for semi-automated WMH segmentation in a slice-by-slice basis using threshold determined by histogram on each slice. Deep and PVWMH identified using morphological image dilation to enlarge CSF. Total WMH was also measured. | N | Y | Not specified | 1.5T  Sequences: T1-weighted FSPGR, T2-weighted and FLAIR | Y |
| 10 | 26 older individuals from the Glostrup 1914 cohort | 80 years | 5 years | Local thresholding automated algorithm. | Modified Scheltens visual rating scale | Y | N | 1.5T  Sequences: T1-, T2-weighted and proton density | Y |
| 11 | 486 patients with symptomatic atherosclerotic disease from the Second Manifestations of ARTerial disease _ MR (SMART-MR) Study | 58 ± 9 years | 3.9 ± 0.4 years | T1-weighted GRE and FLAIR used for brain segmentation. K-nearest-neighbour classification method using voxel intensities, “probabilistically” segments different brain tissues including WMH | N | Y | Y  Based on histogram standardisation | 1.5T  Sequences: T2-weighted, T1 gradient echo-weighted, T2-FLAIR, Inversion recovery (IR) | Not specified |
| 12 | 27 women | 70 years | 18 years (from age 70-88 years), | N | WMH rated in no, mild, moderate and severe. | Y | N | CT scans | Not specified |
| 13 | 639 non-disabled older patients (LADIS Study) | 65 to 84 (74.1± 5) years | 2.42 ± 0.97 years, (median=2.94 years), (yearly assessed) | Semi-automatic delineation by thresholding on each slice using FLAIR | Fazekas visual rating scale | Y | Not reported | 0.5T or 1.5T Sequences: T1-, T2-weighted, FLAIR | Y |
| 14 | 1919 participants of The Cardiovascular Health Study | 65 years and older, mean age 74 years | 5 years | N | Scale 0-9 of white matter worsening using a library of templates | Y | N | Magnet strength not specified.  Sequences: T1-, T2-weighted and spin-density | Y |
| 15 | 1118 older subjects from the 3 Cities study | 72 ± 4.0  (64.9-82.2) years | 4 years | Definition of a common space: average position of the baseline and follow-up images, registered using AIR, WMH assessed automatically using a multispectral 7-class segmentation algorithm. Common WMH, new emerging lesions, classified in PVWMH (3-13 mm from ventricles’ boundaries) and deep (elsewhere). | N | Y | Y  Performed on each slice. Intensity distortion along a line modelled as a 3^rd^ degree polynomial. | 1.5T  Sequences: T1-, T2-weighted, proton density | Y |
| 16 | 13 older individuals from the Cognitive Aging Study ( 1 depressed and 12 depression-free) | 73.5 (mean) ± 4.4 (SD) years | 3 years | semi-automatically by thresholding using a mixture-modelling algorithm (Johnson et al 1993). Used interconnected components to separate periventricular from deep WMH. | N | Y | N | 1.5 T  FLAIR | Not specified |
| 17 | 20 elderly people from (the Rotterdam Scan Study) | 61-88 years (72± 7 years) | 3 years | Semi-automatically by region-growing algorithm and thresholding at one time point but measured twice. Used the mean of both measurements. | 4 visual rating scales: Fazekas scale, Sceltens scale, Rotterdam Scan Study scale and a new scale designed to measure change in WML. | Y | N | 1.5T  T1-, T2-weighted and proton density | Y |
| 18 | 172 patients with relapsing MS and mild disability and 16 controls | Mean age: 36.5 years | 2 years | Volume of T2 lesions, number and volume of enhancing lesions following injection of gadolinium DTPA, volume of T1 hypointensities obtained by thresholding | Visually determined numbers of new and enlarging T2 lesions by comparing MR scans at year 2 with baseline scans | Y | Y (but only used for calculating the brain parenchyma fraction. Method not specified) | Magnet strength not specified  T1-, T2-weighted and proton density | Y |
| 19 | 31 subjects with primary progressive MS within 5 years of disease onset | 26-62 years (mean age 46 years) | 1 year | Lesion loads at baseline estimated from proton density weighted images using Dispimage software and at follow-up by contouring the lesion on the 3DFSPGR scans. | N | Y | Not reported | 1.5T  Only 3D fast spoiled gradient recalled (3D FSPGR)sequence acquired at both time points | Y |
| 20 | 292 participants of the Austrian Stroke Prevention Study | 50-75 (60.2 ± 6.3) years | 3 years and 6 years | Lesion boundaries manually delineated and quantified using Dispimage v4.8 | Fazekas visual rating scale.  Change of WMH in grade and number determined by direct scan comparison | Y | N | 1.5T  Sequences: T1-, T2-weighted and proton density (this last only used for WMH analysis) | Y |
| 21 | 49 non-demented community dwelling individuals (The Oregon Brain Aging Study) | 65 years or older  (84.1± 6.2)years | 10.7±3.2 assessed yearly | Automatically using REGION software (recursive regression analysis) to obtain PVWMH, subcortical and total WMH volumes | N | Y | Not reported | 1.5T  Sequences: T1-, T2-weighted and proton density | Not specified |
| 22 | 21 patients with Relapsing Remitting MS and 10 healthy controls | Mean age 37.5 years | 2 years | In subjects with MS, brain lesion loads determined semi-automatically by local thresholding using Dispimage | N | Y | N | 1.5T  T2-weighted, pre- post-gadolinium enhanced T1-weighted | Y |
| 23 | 100 elderly subjects from the Prospective Study of Pravastatin in the Elderly at Risk (PROSPER) MR imaging Study | 74.5±2.9 years | 33±1.4 months | Semi-automatic using the dual-echo images on a fuzzy inference system, followed by manual editing to remove false positives. | Scheltens visual rating scale | Y | Not reported | 1.5T  Sequences: T2-weighted and proton density | Y (only changed angulations and positioning of the sections) |
| 24 | 665 non-demented patients from the Rotterdam Scan Study | 61.5 ± 5 years | 3.5 years | Used a conventional k-nearest-neighbour brain tissue classifier. For progression, volume on second scan was subtracted from volume on first scan. | N | Y | Y  Nonparametric nonuniform intensity normalisation (N3) | 1.5T  T1-weighted, proton density and FLAIR | Y |
| 25 | 80 ischemic stroke patients | 63.83 (SD=8.72) years | 3 years | Stroke lesions and WMH segmented by thresholding using ANALYZE | N | Y | N | 1.5T  DTI and T2-weighted | Not specified |

Legend: PVWMH: periventricular white matter hyperintensities, FLAIR: Fluid Attenuation Inversion Recovery, DTI: diffusion tensor image, FSPGR: fast spoiled gradient, GRE: gradient echo, CSF: cerebrospinal fluid, IR: inversion recovery

**References:**

1. Bendfeldt K, Kuster P, Traud S, et al: Association of regional gray matter volume loss and progression of white matter lesions in multiple sclerosis – A longitudinal voxel-based morphometry study. Neuroimage, 2009;45:60-67.

2. Callisaya ML, Beare R, Phan TG, Blizzard L et al: Brain structural change and gate decline: a longitudinal population-based study. Am J Geriatr Soc 2013;61:1074-1079.

3. Chen YW, Gurol ME, Rosand J et al: Progression of white matter lesions and haemorrhages in cerebral Amyloid Angiopathy. Neurol 2006; 67:83-87.

4. de Bresser J, Tiehuis AM, van der Berg E et al: Progression of cerebral atrophy and white matter hyperintensities in patients with type 2 diabetes. Diabetes Care 2010; 33(6): 1309-1314.

5. de Leeuw FE, Korf E, Barkhof F, Scheltens P: White matter lesions are associated with progression of medial temporal lobe atrophy in Alzheimer’s Disease. Stroke 2006; 37: 2248-2252.

6. de León M, George AE, Reisberg B, et al: Alzheimer’s disease: longitudinal CT studies of ventricular change. Am J Roentgenol., 1989;152:1257-1262.

7. Dinia L, Bonzano K, Albano B et al: White matter lesions progression in migraine with aura: a clinical and MRI longitudinal study. J Neuroimaging 2013; 23: 47-52.

8. Dufouil C, de Kersaint-Gilly A, Besancon V, et al: Longitudinal study of blood pressure and white matter hyperintensities : the EVA MRI Cohort. Neurology, 2001;56:921-926.

9. Firbank MJ, Wiseman RM, Burton EJ et al: Brain atrophy and white matter hyperintensity change in older adults and relationship to blood pressure. J Neurol 2007; 254:713-721.

10. Garde E, Lykke Mortensen E, Rostrup E, Paulson OB: Decline in intelligence is associated with white matter hyperintensity volume. J Neurol Neurosurg Psychiatry, 2005;76:1289-1291.

11. Grool AM, van der Graaf Y, Witkamp TD et al: Progression of white matter lesion volume and health-related quality of life in patients with symptomatic atherosclerotic disease: the SMART-MR study. J Ag Res 2011

12. Gustafson DR, Steen B, Skoog I: Body mass index and white matter lesions in elderly women. An 18-year longitudinal study. Intl Psychogeriatrics, 2004;16(3):327-336.

13. Inzitari D, Pracucci G, Poggesi A, Carlucci G, Barkhof F, Chabriat H, Erkinjuntti T, Fazekas F, Ferro JM, Hennerici M, Langhorne P, O’Brien J, Scheltens P, Visser MC, Wahlund LO, Waldemar G, Wallin A and Pantoni L on behalf of the LADIS Study Group: Changes in white matter as determinant of global functional decline in older independent outpatients: three year follow-up of LADIS (leukoaraiosis and disability) study cohort. BMJ, 2009;339:b2477:279-282.

14. Longstreth WT, Arnold AM, Beauchamp NJ, Manolio TA, Lefkowitz D, Jungreis C, Hirsch, CH, O’Leary DH and Furberg CD: Incidence, manifestations and predictors of worsening white matter on serial cranial Magnetic Resonance Imaging in the elderly: The Cardiovascular Health Study. Stroke, 2005;36:56-61.

15. Maillard P, Crivello F, Dufouil C, Tzourio-Mazoyer N, Tzourio C, Mazoyer B: Longitudinal follow-up of individual white matter hyperintensities in a large cohort of elderly. Neuroradiology, 2009;51:209-220.

16. Nebes RD, Reynolds CF, Boada F, et al: Longitudinal increase in the volume of white matter hyperintensities in late-onset depression. Int J Geriatr Psychiatry, 2002;17:526-530.

17. Prins ND, van Straaten EC, van Dijk EJ et al. Measuring progression of cerebral white matter lesions on MRI: visual ratings and volumetrics. Neurology 2004;62:1533-1539.

18. Rudick RA, Fisher E, Lee JC, et al: Use of the brain perenchymal fraction to measure whole brain atrophy in relapsing-remitting MS. Neurology, 1999;53:1698-1704.

19. Sastre-Garriga J, Ingle GT, Chard DT, et al: Grey and white matter volume changes in early primary progressive multiple sclerosis: a longitudinal study. Brain, 2005;128:1454-1460.

20. Schmidt R, Enzinger C, Ropele S, Schmidt H, Fazekas F: Progression of cerebral white matter lesions: 6 years results of the Austrian Stroke Prevention Study. Lancet, 2003;361:2046-2048.

21. Silbert LC, Howieson DB, Dodge H and Kaye JA: Cognitive impairment risk: White matter hyperintensity progression matters. Neurology, 2009; 73;120-125.

22. Tiberio M, Chard DT, Altmann DR, et al: Gray and white matter volume changes in early RRMS: a 2-year longitudinal study. Neurology, 2005;64:1001-1007.

23. van den Heuvel DMJ, ten Dam VH, de Craen AJM, Admiraal-Behloul F, van Es AGCM, Palm WM, Spilt A, Bollen ELEM, Blauw GJ, launer L, et al. Measuring longitudinal white matter changes: comparison of a visual rating scale with a volumetric measurement. AJNR 2006;27:875-878.

24. Verhaaren BFG, Vernooij MW, de Boer R et al: High blood pressure and cerebral white matter lesion progression in the general population. Hypertension 2013; 61:1354-1359.

25. Wang C, Stebbins GT, Nyenhuis DL, et al: Longitudinal changes in white matter following ischemic stroke: A three-year follow-up study. Neurobiol. of Aging, 2006;27:1827-1833.
