# Supplementary material for: On the computational assessment of white matter hyperintensity progression: difficulties in method selection and bias field correction performance on images with significant white matter pathology
Source: Neuroradiology. 2016 Jan 30;58:475–85. doi: 10.1007/s00234-016-1648-3 (PMC4846712; doi:10.1007/s00234-016-1648-3)
Supplement: Supplementary file 3 — (DOCX 16 kb) [file 234_2016_1648_MOESM3_ESM.docx]

**On the computational assessment of white matter hyperintensity progression: difficulties in method selection and bias field correction performance on images with significant white matter pathology**

**Supplementary Table S2.** Differences between variance-to-mean ratios (ΔVMR) of intensities on normal-appearing tissues (i.e. excluding hyperintensities) expressed in median (IQR). These were obtained from different scenarios (first column from left to right), by the following 4 equations:

ΔVMR_baseline_ = (SD^2^_original_ / Mean_original_) – (SD^2^_corrected_ / Mean_corrected_) (1)

ΔVMR_follow-up_ = (SD^2^_original_ / Mean_original_) – (SD^2^_corrected_ / Mean_corrected_) (2)

ΔVMR_original_ = (SD^2^_follow-up_/ Mean_follow-up_) – (SD^2^_baseline_ / Mean_baseline_) (3)

ΔVMR _corrected_= (SD^2^_follow-up_/ Mean_follow-up_) – (SD^2^_baseline_ / Mean_baseline_) (4)

where SD (or Mean)_original_ refers to the standard deviation (or Mean intensity) on the normal tissues of the original image and SD (or Mean)_corrected_ refers to those of the bias field corrected (BFC) image.

| BFC method | Variation analysed | Image in which the variation was calculated | FLAIR | T2*-weighted | T2-weighted |
| --- | --- | --- | --- | --- | --- |
| N4 applied to original images | BFC vs. uncorrected | baseline (ΔVMR_baseline_) | 1.36 (0.98) | 1.94 (1.83) | 4.25 (1.93) |
|  |  | follow-up  (ΔVMR_follow-up_) | 2.56 (0.78) | 4.20 (1.67) | 16.35 (7.45) |
|  | Significance (p) | baseline vs. follow-up | < 0.001 | | |
|  | Baseline vs. follow-up | BFC  (ΔVMR_corrected_) | 2.27 (2.80) | 3.17 (3.77) | 113.40 (63.27) |
|  |  | Uncorrected  (ΔVMR_original_) | 3.40 (4.36) | 4.60 (6.37) | 126.28 (64.64) |
|  | Significance (p) | BFC vs. uncorrected | < 0.001 | | |
| N4 applied to ICV extracted images | BFC vs. uncorrected | baseline  (ΔVMR_baseline_) | 0.30 (0.53) | 1.25 (2.14) | 3.19 (4.98) |
|  |  | follow-up (ΔVMR_follow-up_) | 0.48 (1.48) | 2.58 (4.00) | 3.17 (27.18) |
|  | Significance (p) | baseline vs. follow-up | 0.481 | 0.596 | 0.074 |
|  | Baseline vs. follow-up | BFC  (ΔVMR_corrected_) | 3.29 (3.83) | 4.15 (4.08) | 132.34 (80.51) |
|  |  | uncorrected (ΔVMR_original_) | 3.40 (4.37) | 4.60 (6.37) | 126.28 (64.64) |
|  | Significance (p) | BFC vs. uncorrected | 0.481 | 0.596 | 0.074 |
| N4 applied after extracting the ICV and masking the stroke lesion | BFC vs. uncorrected | aseline  (ΔVMR_baseline_) | 0.28 (0.49) | 1.21 (2.20) | 3.22 (5.12) |
|  |  | follow-up (ΔVMR_follow-up_) | 0.53 (1.60) | 2.68 (4.00) | 4.34 (27.10) |
|  | Significance (p) | baseline vs. follow-up | 0.385 | 0.385 | 0.091 |
|  | Baseline vs. follow-up | BFC  (ΔVMR_corrected_) | 3.30 (3.81) | 4.11 (4.25) | 132.34 (78.38) |
|  |  | uncorrected (ΔVMR_original_) | 3.40 (4.37) | 4.60 (6.37) | 126.28 (64.64) |
|  | Significance (p) | BFC vs. uncorrected | 0.385 | 0.385 | 0.091 |
| FSL-FAST applied to original images | BFC vs. uncorrected | baseline  (ΔVMR_baseline_) | 0.83 (1.30) | 1.37 (1.98) | 2.78 (3.74) |
|  |  | follow-up (ΔVMR_follow-up_) | 2.27 (0.80) | 3.39 (1.12) | 36.28 (15.79) |
|  | Significance (p) | baseline vs. follow-up | < 0.001 | | |
|  | Baseline vs. follow-up | BFC  (ΔVMR_corrected_) | 2.08 (2.39) | 2.93 (4.17) | 98.94 (49.10) |
|  |  | uncorrected (ΔVMR_original_) | 3.40 (4.37) | 4.60 (6.37) | 126.28 (64.64) |
|  | Significance (p) | BFC vs. uncorrected | < 0.001 | | |
| FSL-FAST applied to ICV extracted images | BFC vs. uncorrected | baseline  (ΔVMR_baseline_) | 0.49 (0.78) | 0.94 (1.07) | 1.22 (2.03) |
|  |  | follow-up (ΔVMR_follow-up_) | 1.44 (0.69) | 1.77 (0.88) | 8.08 (5.37) |
|  | Significance (p) | baseline vs. follow-up | < 0.001 | | |
|  | Baseline vs. follow-up | BFC  (ΔVMR_corrected_) | 2.59 (3.15) | 4.09 (4.38) | 121.14 (60.16) |
|  |  | uncorrected (ΔVMR_original_) | 3.40 (4.37) | 4.60 (6.37) | 126.28 (64.64) |
|  | Significance (p) | BFC vs. uncorrected | < 0.001 | | |
| FSL-FAST applied after extracting the ICV and masking the stroke lesion | BFC vs. uncorrected | baseline  (ΔVMR_baseline_) | 0.49 (0.78) | 0.94 (1.07) | 1.22 (2.03) |
|  |  | follow-up (ΔVMR_follow-up_) | 1.44 (0.65) | 1.78 (0.89) | 8.11 (5.40) |
|  | Significance (p) | baseline vs. follow-up | < 0.001 | | |
|  | Baseline vs. follow-up | BFC  (ΔVMR_corrected_) | 2.58 (3.14) | 4.06 (4.37) | 121.10 (60.14) |
|  |  | uncorrected (ΔVMR_original_) | 3.40 (4.37) | 4.60 (6.37) | 126.28 (64.64) |
|  | Significance (p) | BFC vs. uncorrected | < 0.001 | | |
| E^2^D-HUM applied to original images | BFC vs. uncorrected | baseline  (ΔVMR_baseline_) | 0.17 (0.33) | -0.02 (0.48) | -0.72 (0.92) |
|  |  | follow-up (ΔVMR_follow-up_) | 0.51 (0.21) | 0.03 (0.53) | -3.42 (2.67) |
|  | Significance (p) | baseline vs. follow-up | <0.001 | 0.943 | <0.001 |
|  | Baseline vs. follow-up | BFC  (ΔVMR_corrected_) | 3.18 (4.07) | 4.82 (5.96) | 130.62 (64.48) |
|  |  | uncorrected (ΔVMR_original_) | 3.40 (4.37) | 4.60 (6.37) | 126.28 (64.64) |
|  | Significance (p) | BFC vs. uncorrected | < 0.001 | 0.943 | <0.001 |
| E^2^D-HUM applied to ICV extracted images | BFC vs. uncorrected | baseline  (ΔVMR_baseline_) | 0.08 (0.10) | 0.23 (0.29) | 0.23 (0.23) |
|  |  | follow-up (ΔVMR_follow-up_) | 0.16 (0.07) | 0.39 (0.47) | 0.92 (0.96) |
|  | Significance (p) | baseline vs. follow-up | 0.003 | <0.001 | |
|  | Baseline vs. follow-up | BFC  (ΔVMR_corrected_) | 3.30 (4.23) | 4.40 (5.45) | 126.01 (63.97) |
|  |  | uncorrected (ΔVMR_original_) | 3.40 (4.37) | 4.60 (6.37) | 126.28 (64.64) |
|  | Significance (p) | BFC vs. uncorrected | 0.003 | <0.001 | |
| E^2^D-HUM applied after extracting the ICV and masking the stroke lesion | BFC vs. uncorrected | baseline  (ΔVMR_baseline_) | 0.08 (0.11) | 0.23 (0.29) | 0.24 (0.23) |
|  |  | follow-up (ΔVMR_follow-up_) | 0.16 (0.08) | 0.39 (0.47) | 0.92 (0.97) |
|  | Significance (p) | baseline vs. follow-up | 0.002 | <0.001 | |
|  | Baseline vs. follow-up | BFC  (ΔVMR_corrected_) | 3.30 (4.24) | 4.39 (5.45) | 126.01 (63.98) |
|  |  | uncorrected (ΔVMR_original_) | 3.40 (4.37) | 4.60 (6.37) | 126.28 (64.64) |
|  | Significance (p) | BFC vs. uncorrected | 0.002 | <0.001 | |
