# Supplementary figures and images for: On the computational assessment of white matter hyperintensity progression: difficulties in method selection and bias field correction performance on images with significant white matter pathology
Source: Neuroradiology. 2016 Jan 30;58:475–85. doi: 10.1007/s00234-016-1648-3 (PMC4846712; doi:10.1007/s00234-016-1648-3)

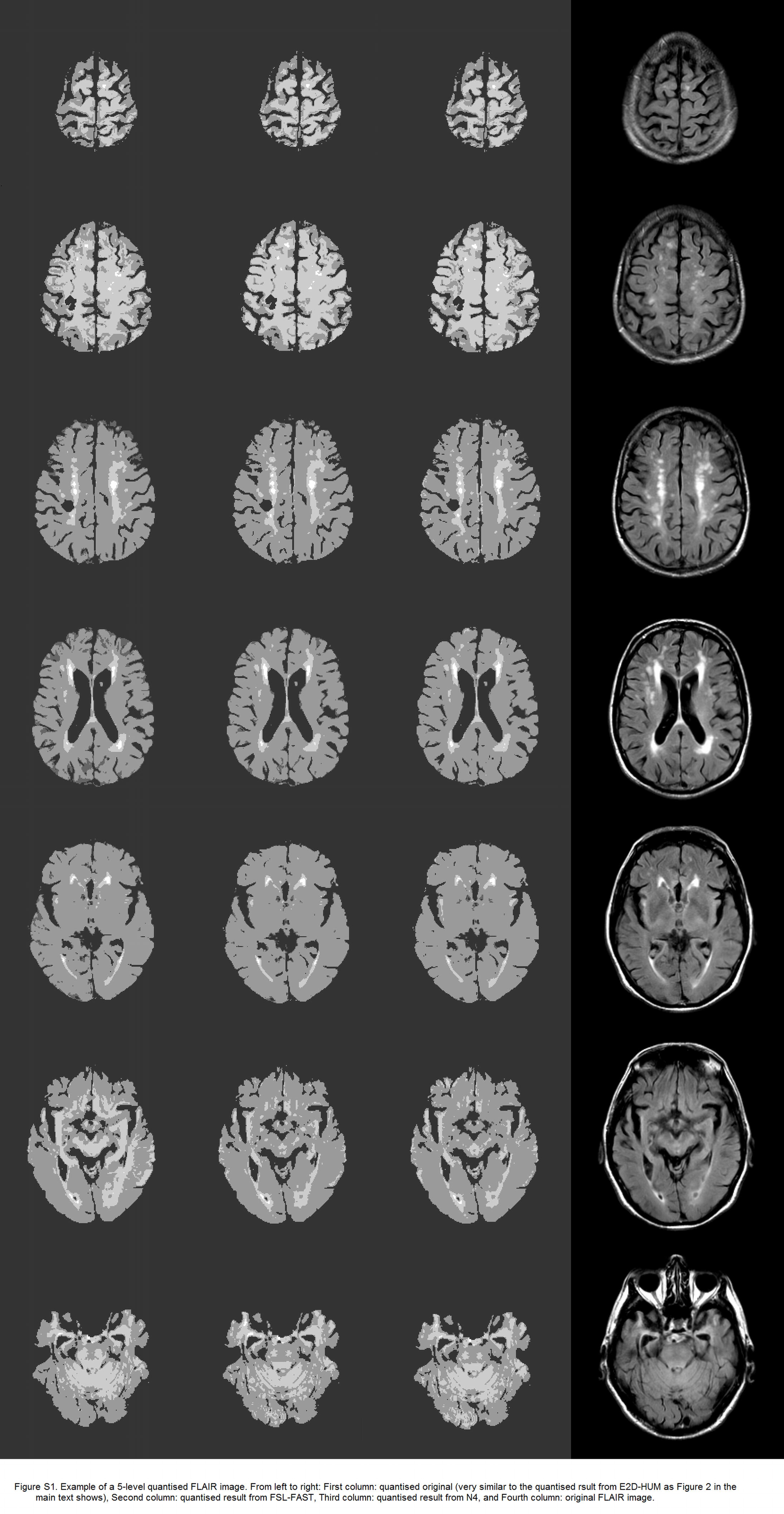

Supplement: Supplementary file 4 — (JPG 644 kb) [file 234_2016_1648_Fig5_ESM.jpg]

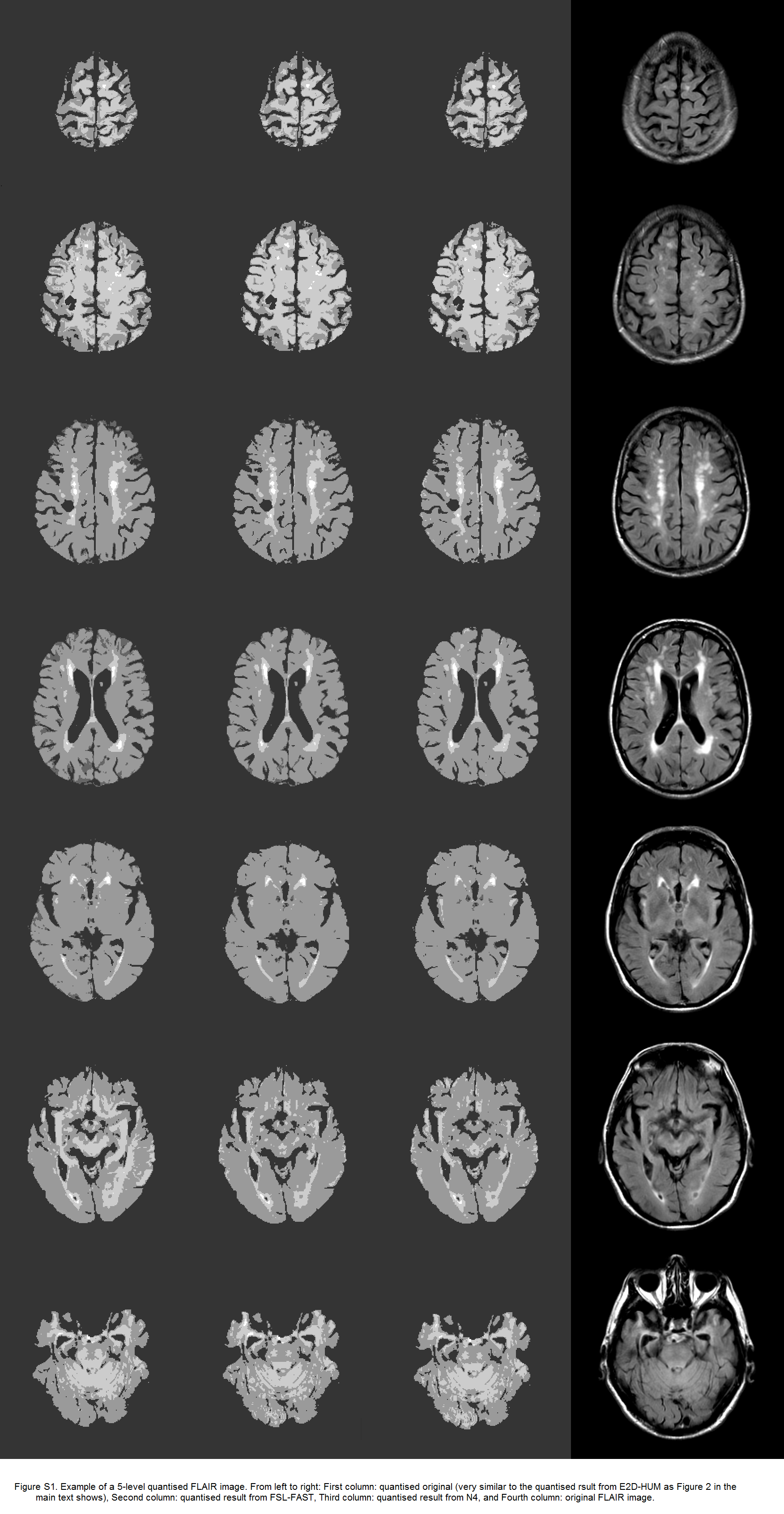

Supplement: Supplementary file 5 — High Resolution Image (TIF 1648 kb) [file 234_2016_1648_MOESM4_ESM.tif]

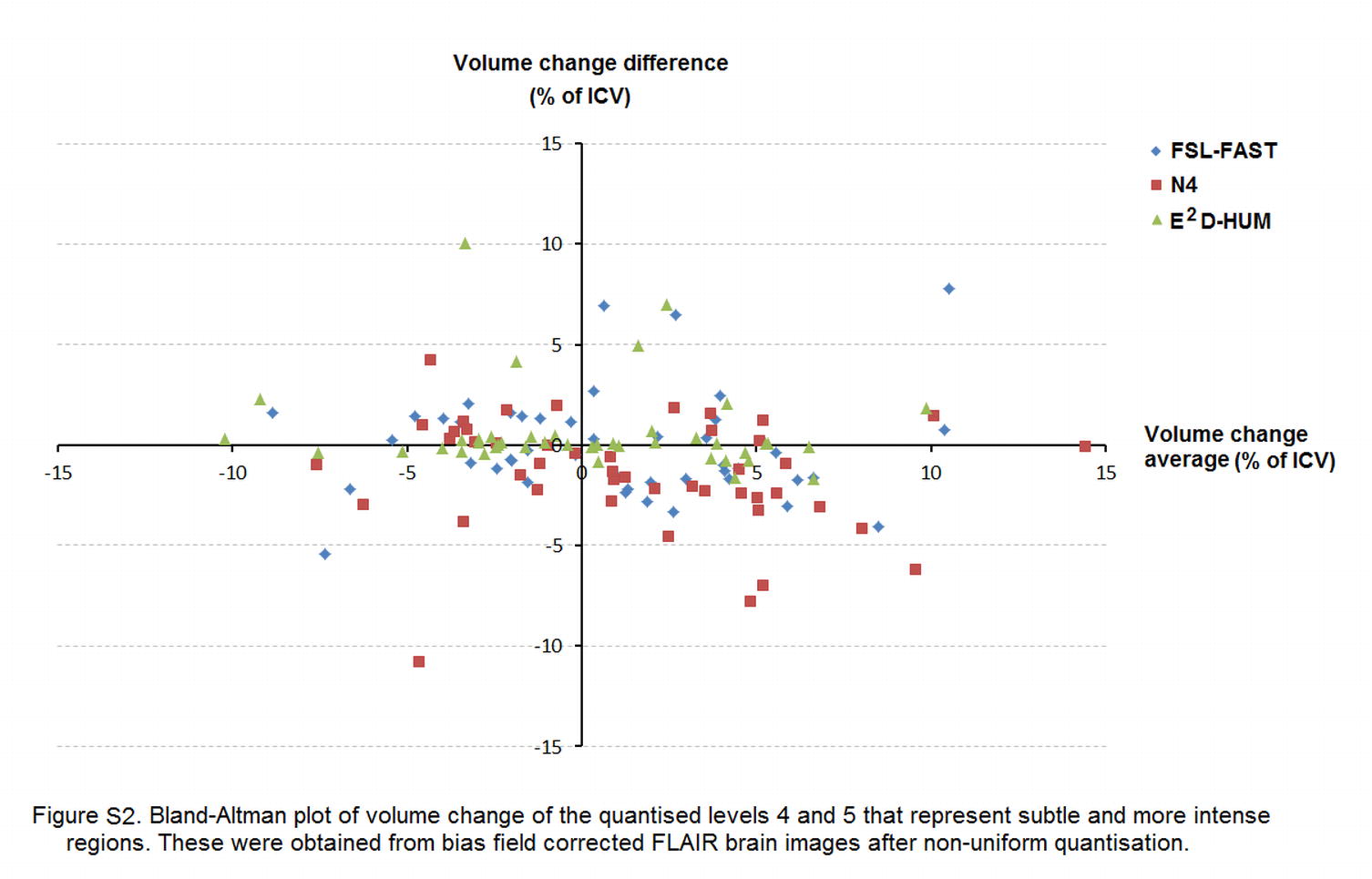

Supplement: Supplementary file 6 — (JPG 93 kb) [file 234_2016_1648_Fig6_ESM.jpg]

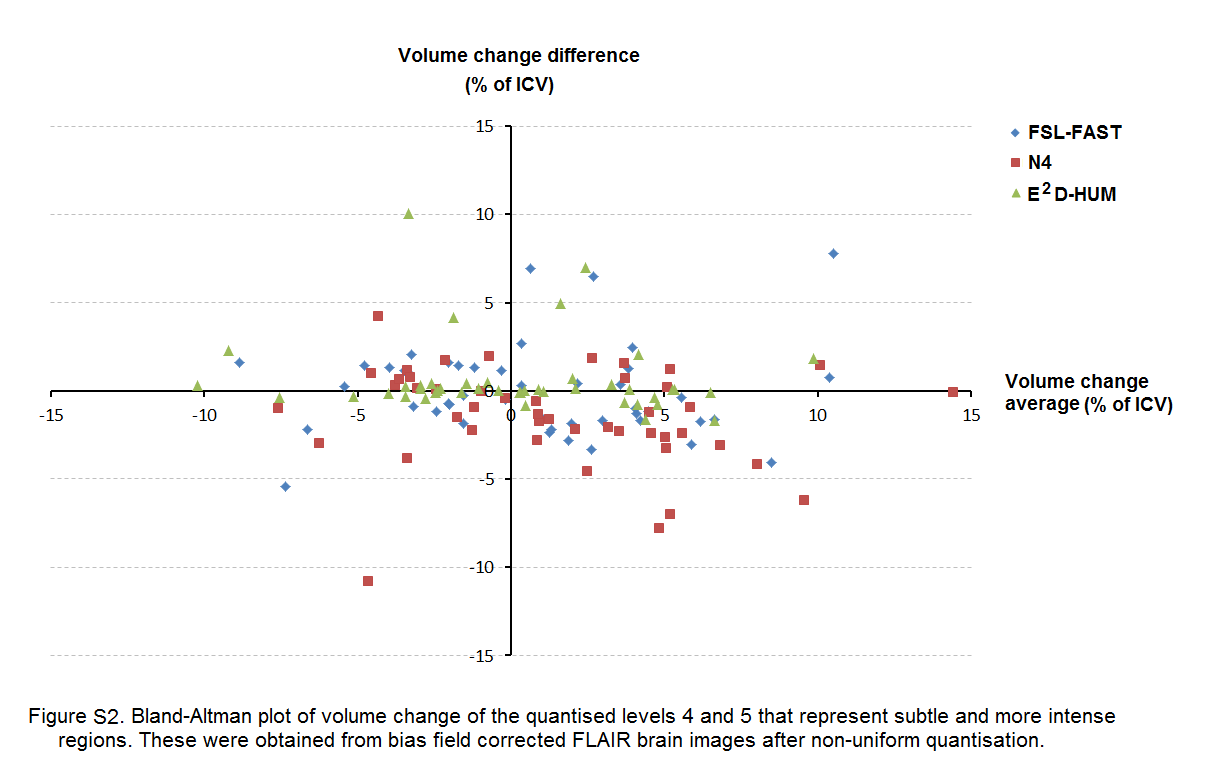

Supplement: Supplementary file 7 — High Resolution Image (TIF 122 kb) [file 234_2016_1648_MOESM5_ESM.tif]

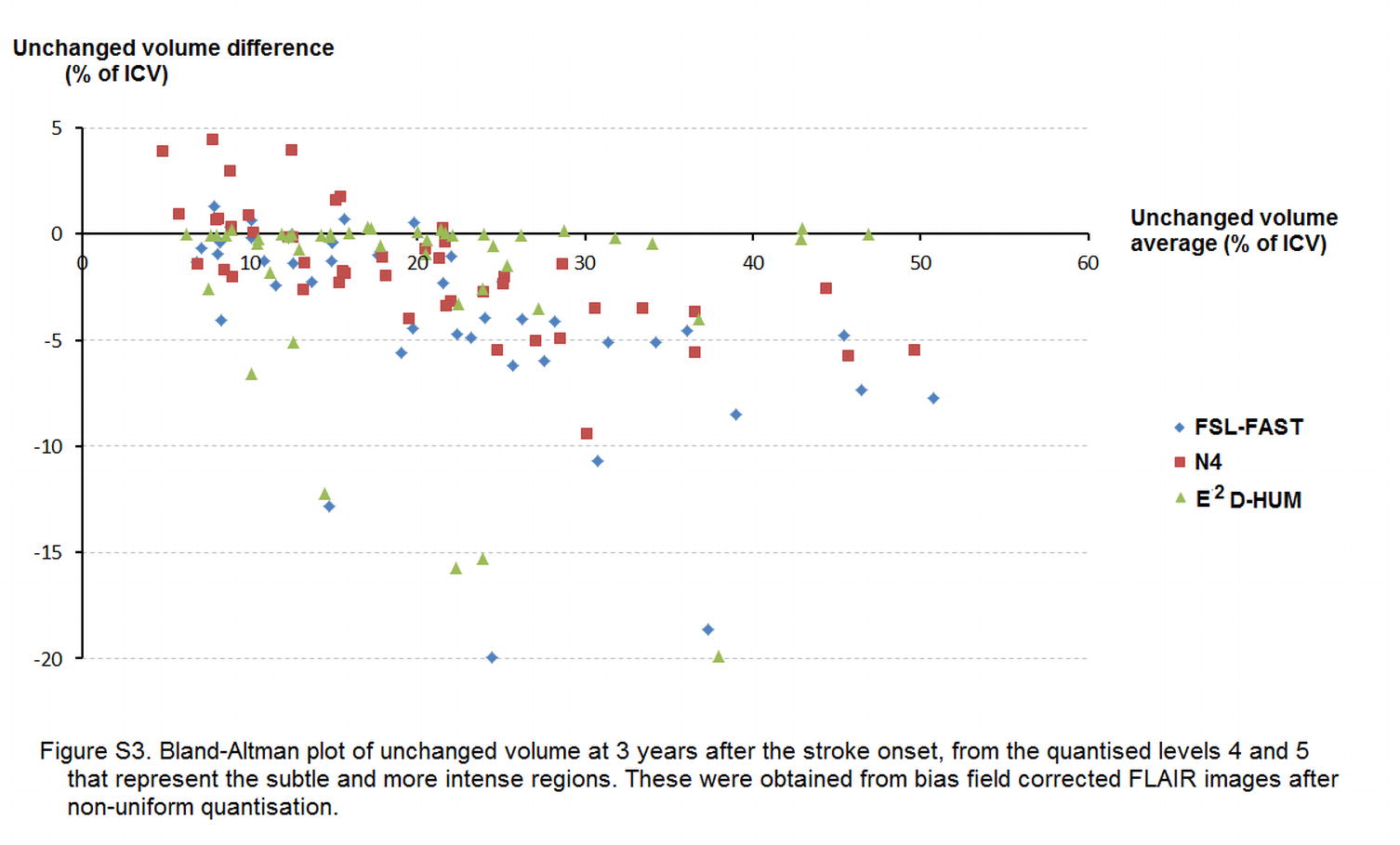

Supplement: Supplementary file 8 — (JPG 99 kb) [file 234_2016_1648_Fig7_ESM.jpg]

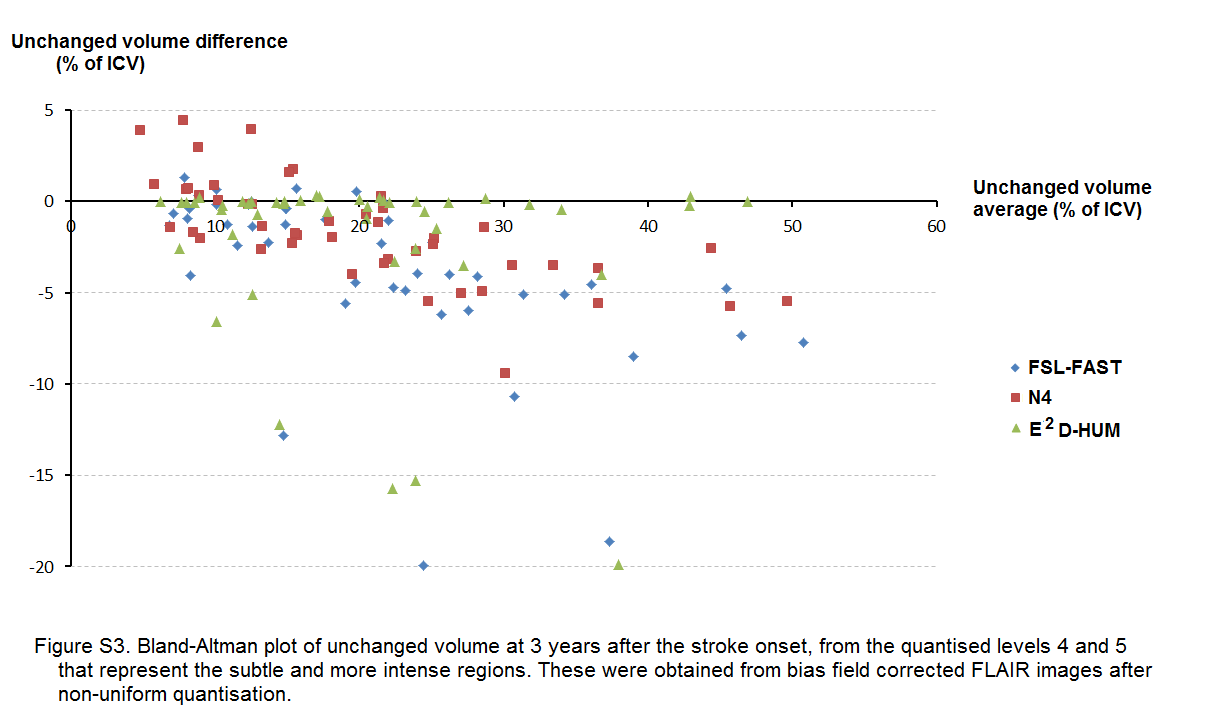

Supplement: Supplementary file 9 — High Resolution Image (TIF 122 kb) [file 234_2016_1648_MOESM6_ESM.tif]

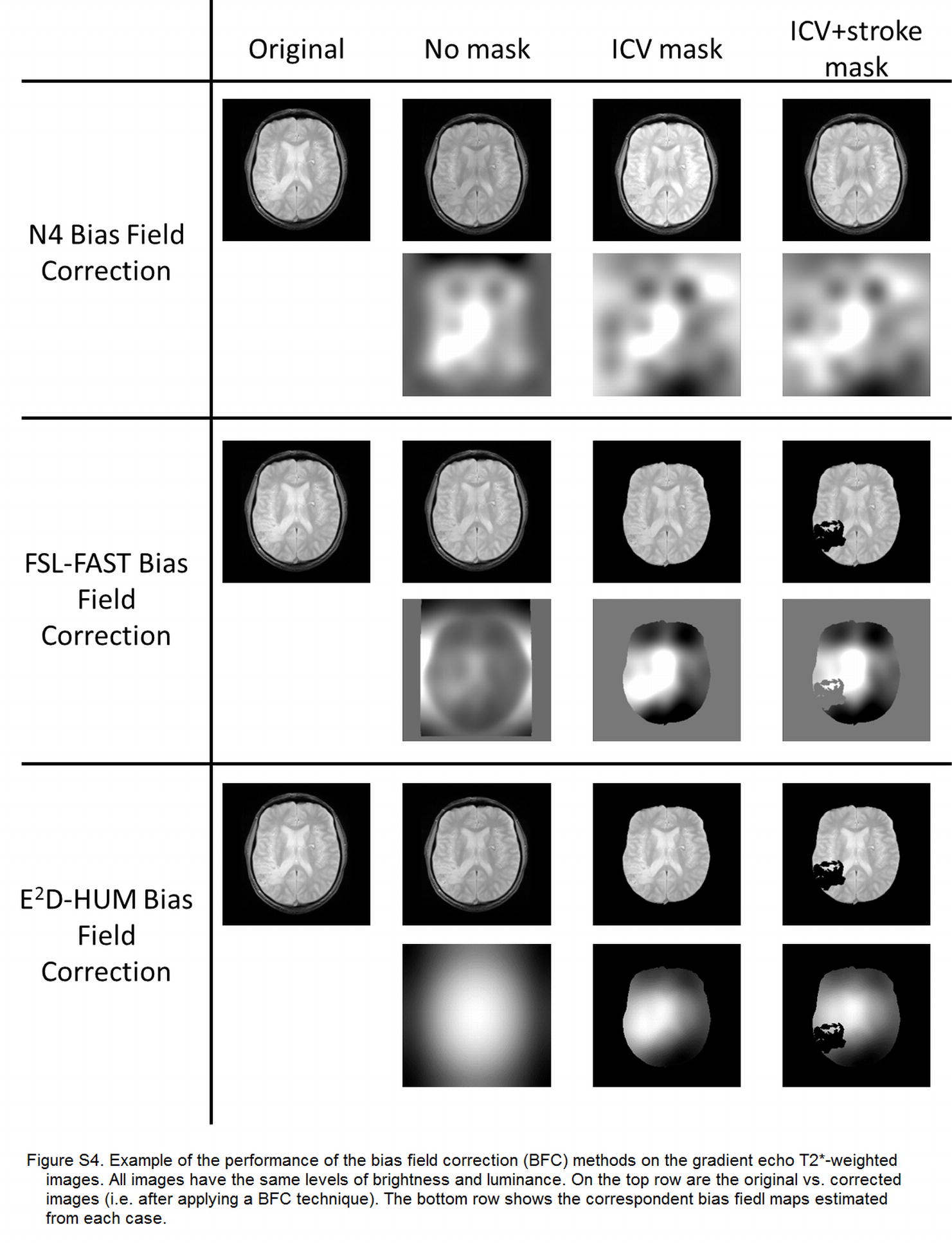

Supplement: Supplementary file 10 — (JPG 223 kb) [file 234_2016_1648_Fig8_ESM.jpg]

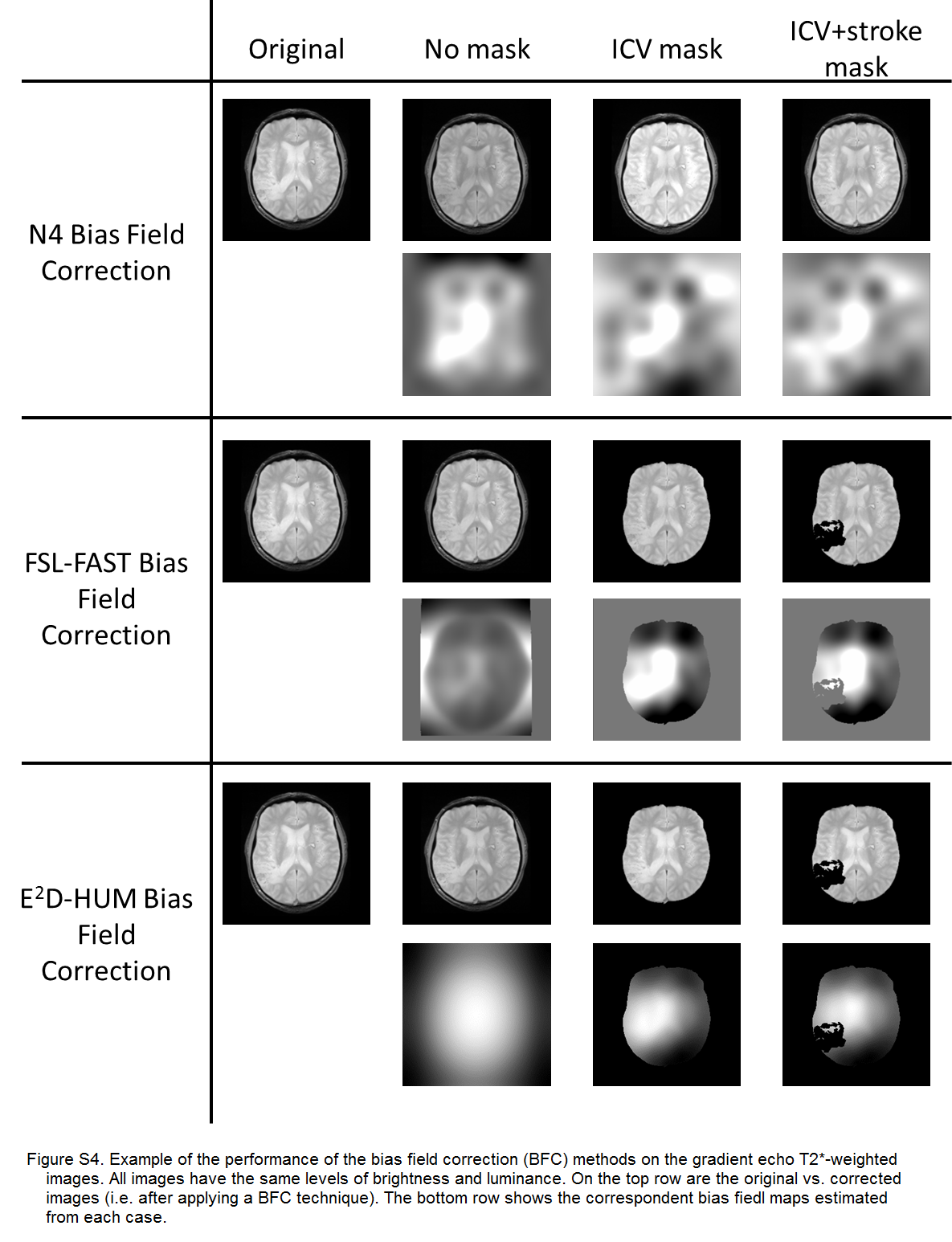

Supplement: Supplementary file 11 — High Resolution Image (TIF 727 kb) [file 234_2016_1648_MOESM7_ESM.tif]

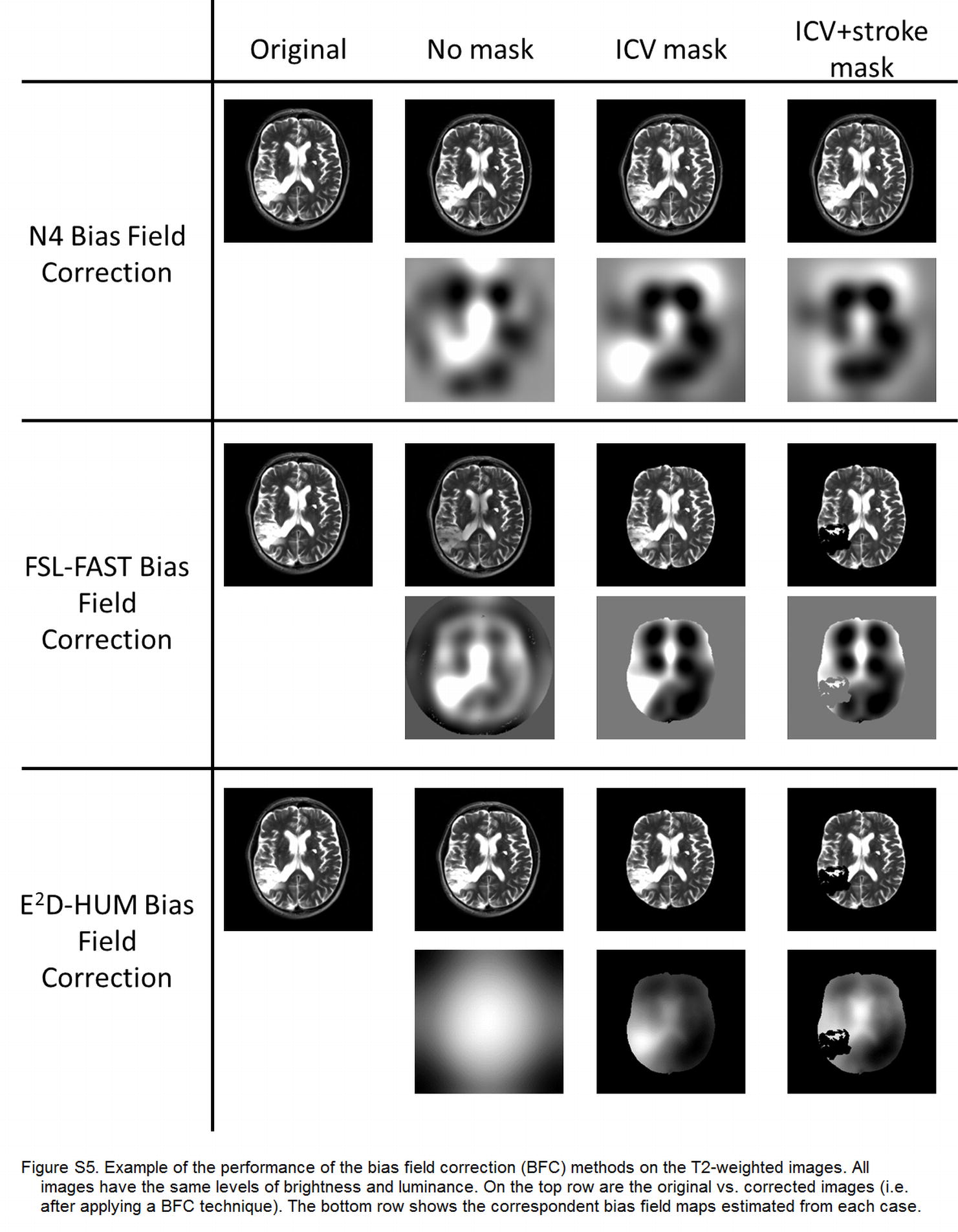

Supplement: Supplementary file 12 — (JPG 244 kb) [file 234_2016_1648_Fig9_ESM.jpg]

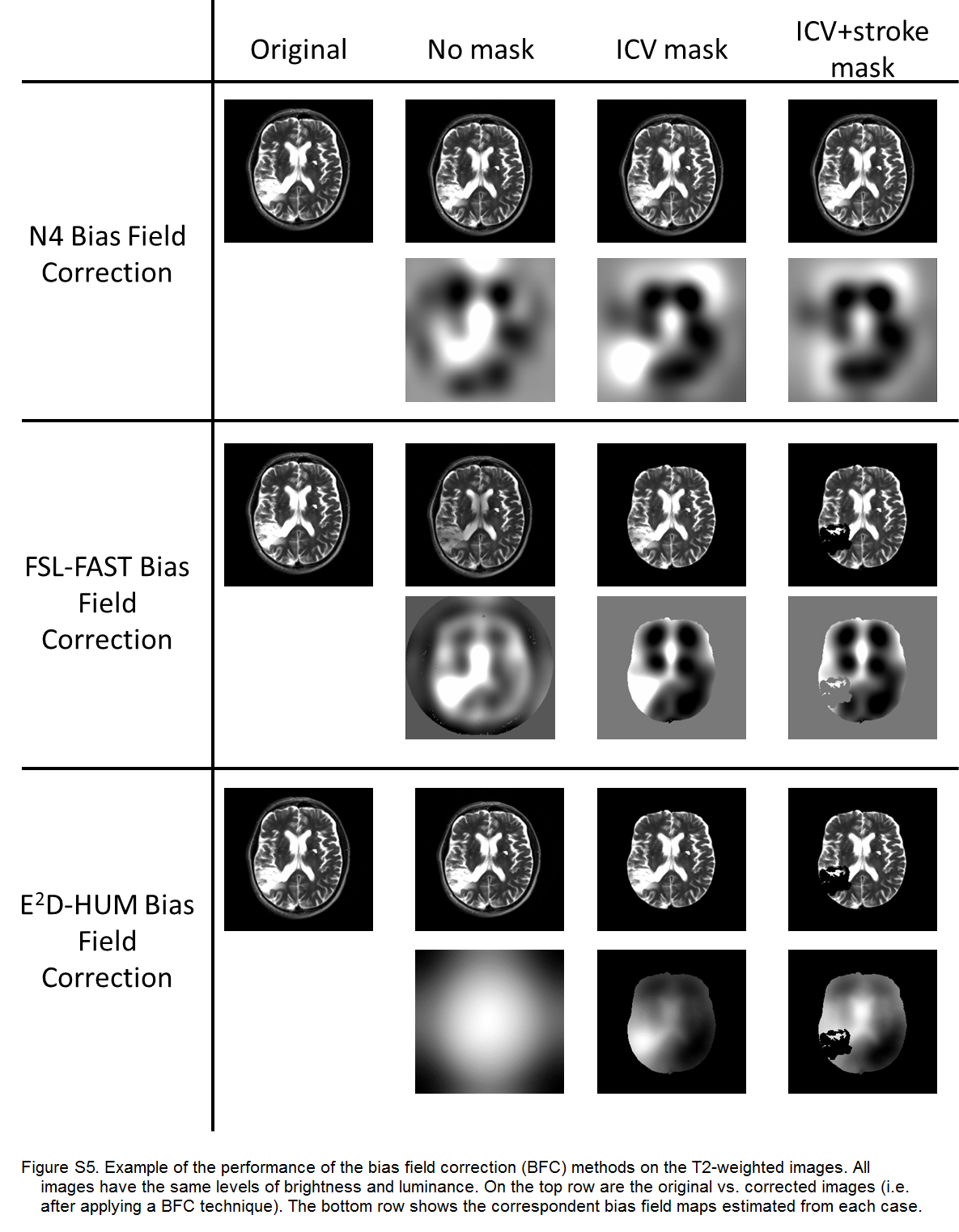

Supplement: Supplementary file 13 — High Resolution Image (TIF 780 kb) [file 234_2016_1648_MOESM8_ESM.tif]

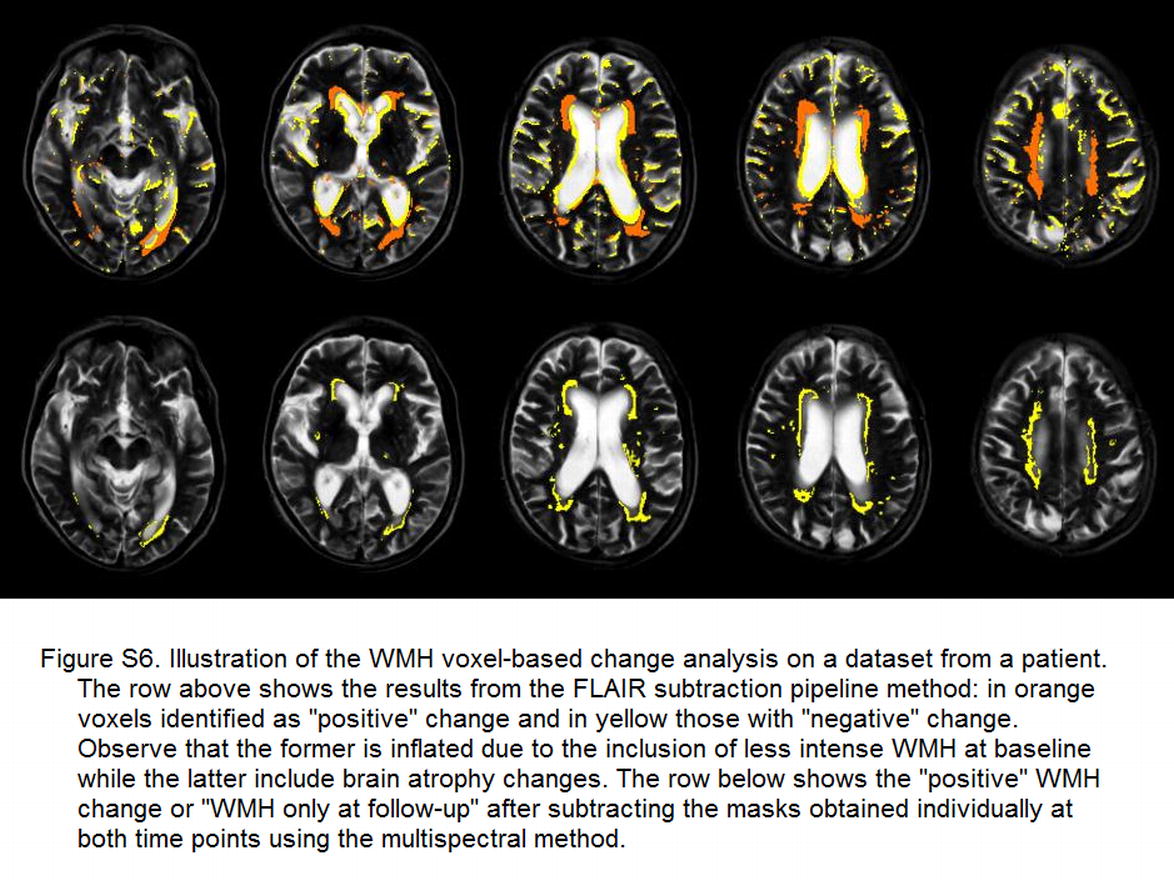

Supplement: Supplementary file 14 — (JPG 179 kb) [file 234_2016_1648_Fig10_ESM.jpg]

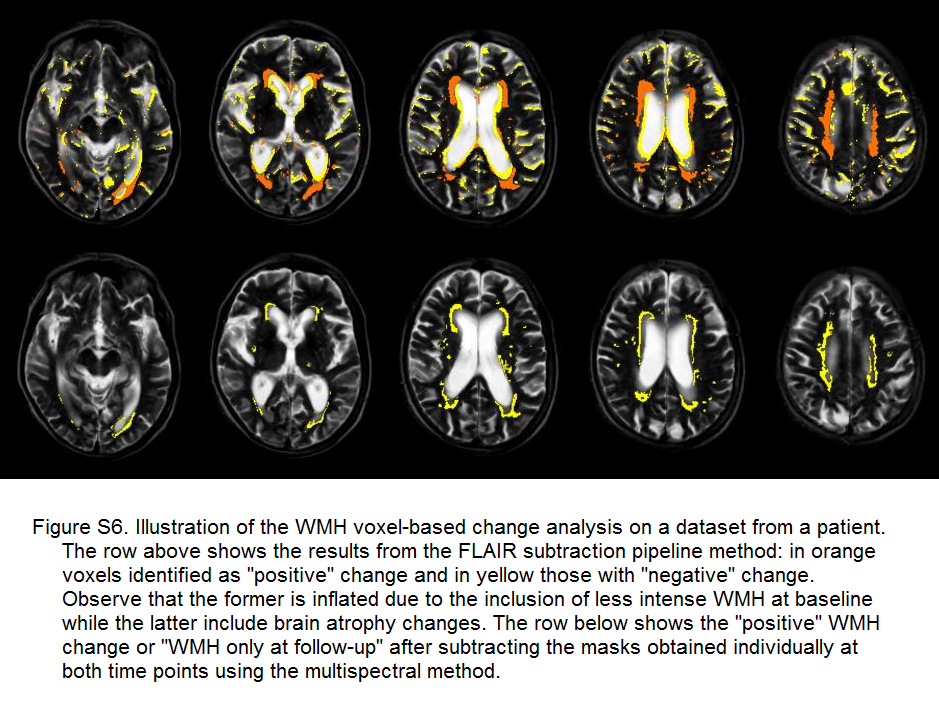

Supplement: Supplementary file 15 — High Resolution Image (TIF 700 kb) [file 234_2016_1648_MOESM9_ESM.tif]
